# Supplementary figures and images for: A male-killing Wolbachia endosymbiont is concealed by another endosymbiont and a nuclear suppressor
Source: PLoS Biol. 2023 Mar 22;21(3):e3001879. doi: 10.1371/journal.pbio.3001879 (PMC10069767; doi:10.1371/journal.pbio.3001879)

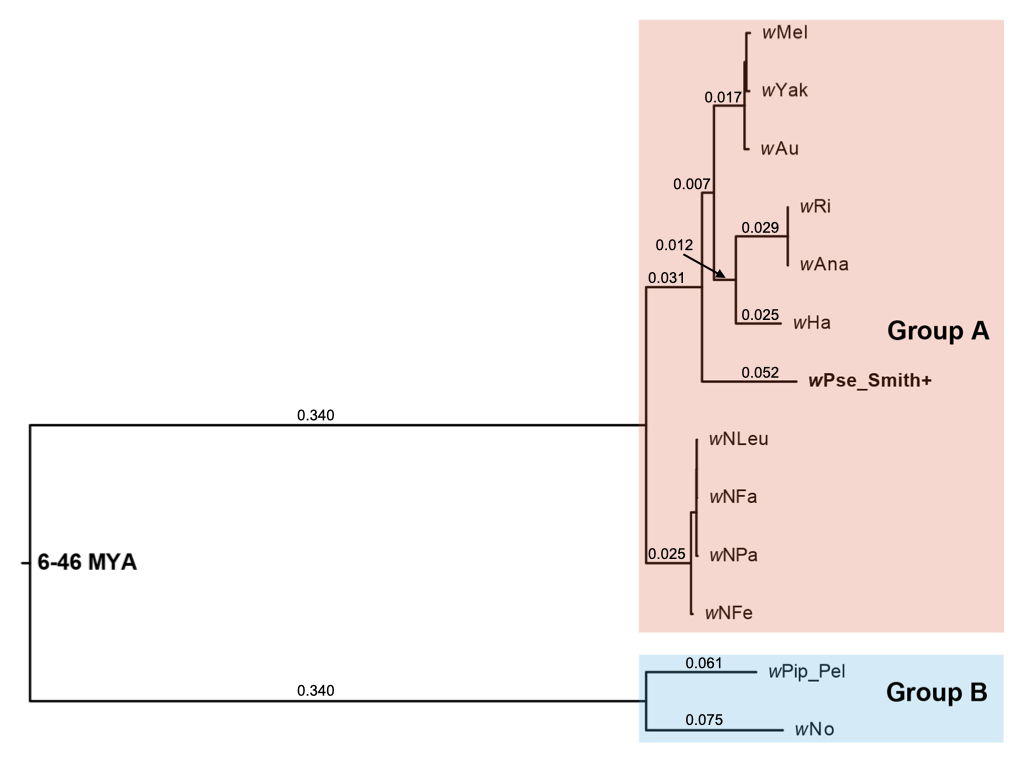

Supplement: S1 Fig — An estimated Bayesian phylogram for various Group-A (red) and Group-B (blue) Wolbachia strains. wPse Smith+ is a Group-A strain and outgroup to a larger clade containing wHa, wRi-like, and wMel-like strains. The 4 Wolbachia infecting Nomada bees (wNFe, wNPa, wNLeu, and wNFa) are outgroup to the clade containing wPse Smith+. These Group-A Wolbachia diverged from Group-B Wolbachia (wPip_Pel and wNo) up to 46MYA (divergence time superimposed from Meany and colleagues [37]). The phylogram was estimated with 168 genes and a total of 136,545 bp. Nodes with posterior probability <0.95 were collapsed into polytomies. The sum of all branch lengths was fixed to one. Very small branch lengths (i.e., = /< 0.003) are excluded to improve figure readability. The data underlying this figure can be found in https://doi.org/10.26188/21892974.v1. (TIFF) [file pbio.3001879.s001.tiff]

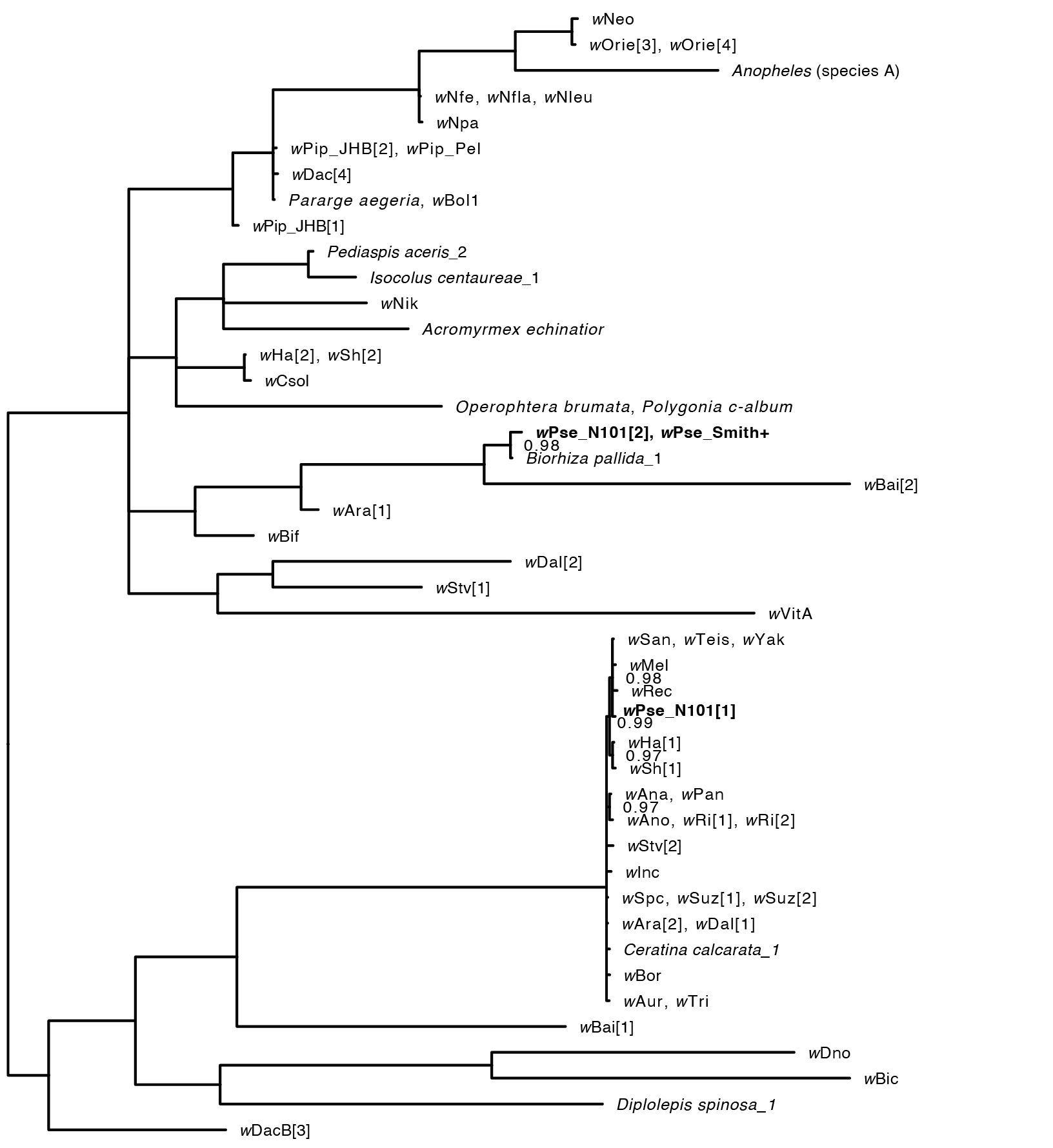

Supplement: S3 Fig — Identical samples were collapsed into a single tip, and nodes with posterior probability <0.95 were collapsed into polytomies. The root position is not known, but the tree was midpoint rooted for legibility. cifAwPse_N101[2] is identical to cifAwPse_Smith+ and sister to the cifA[T1] set observed in the genome of the unnamed Wolbachia variant that infects gall wasp Biorhiza pallida. cifAwPse_N101[1], cifAwMel, cifAwRec, cifAwSan, cifAwTeis, and cifAwYak comprise a polytomy that is sister to a clade containing cifAwHa and cifAwSh, assuming that the true root does not fall within this focal clade. We only report the posterior probability node support values that are less than 1. The data underlying this figure can be found in https://doi.org/10.26188/21892974.v1. (TIF) [file pbio.3001879.s003.tif]

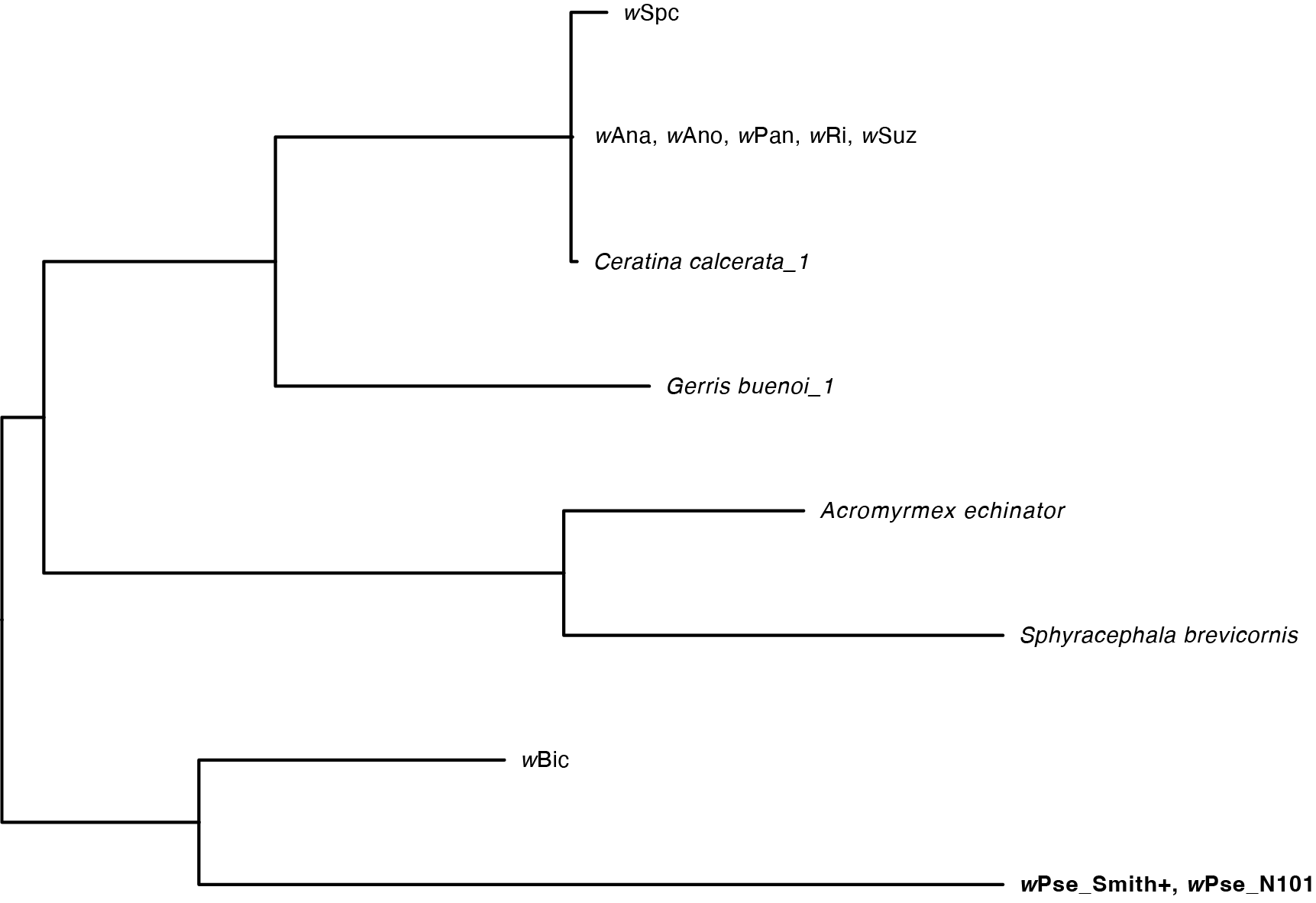

Supplement: S4 Fig — Identical samples were collapsed into a single tip, and nodes with posterior probability <0.95 were collapsed into polytomies. The root position is not known, but the tree was midpoint rooted for legibility. cifAwPse_Smith+ and cifAwPse_N101 copies are identical and sister to cifAwBic from Drosophila bicornuta, assuming the true root does not fall in this clade. All nodes shown have a posterior probability support value of 1. The data underlying this figure can be found in https://doi.org/10.26188/21892974.v1. (TIF) [file pbio.3001879.s004.tif]

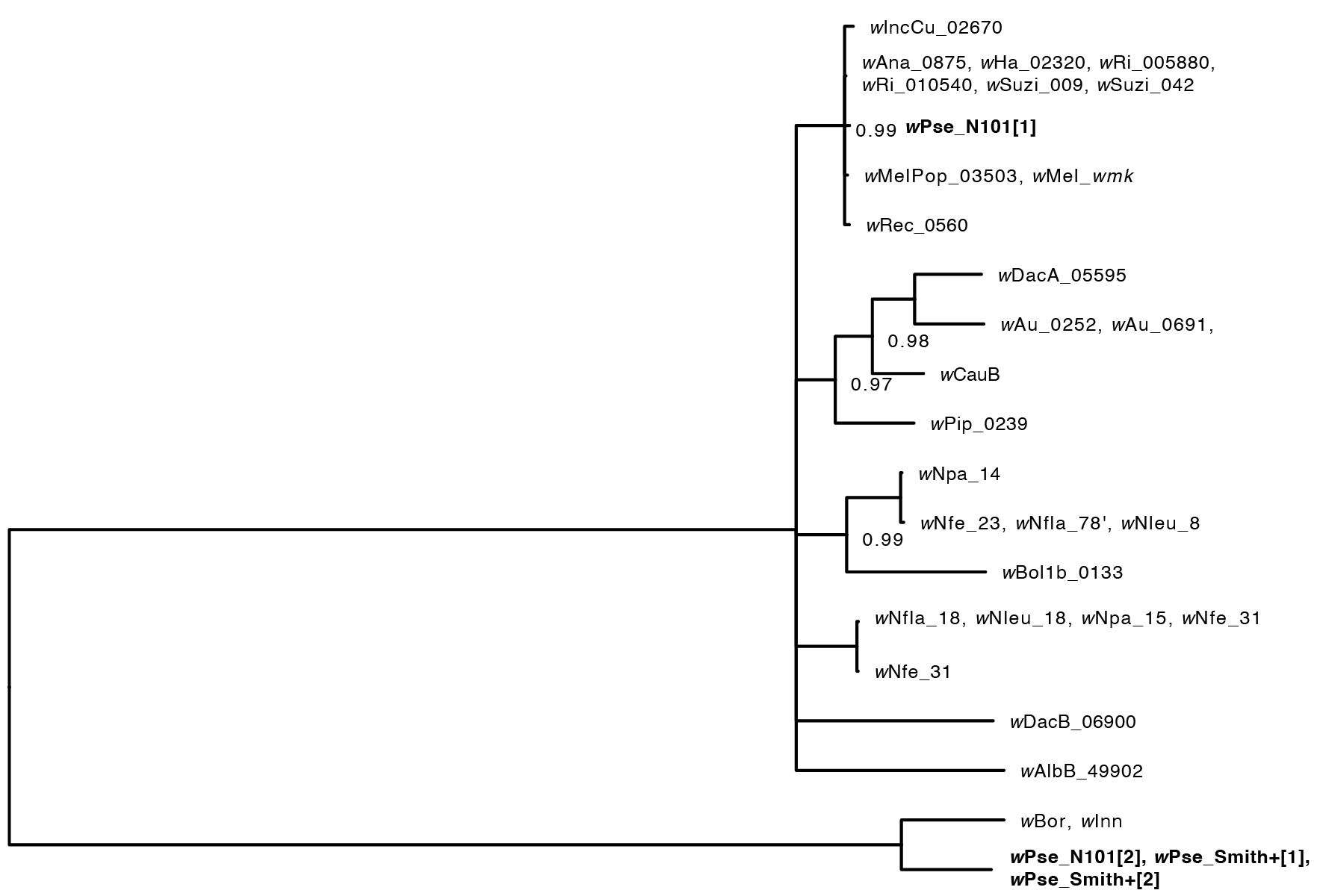

Supplement: S5 Fig — Identical samples were collapsed into a single tip, and nodes with posterior probability <0.95 were collapsed into polytomies. The root position is not known, but the tree was midpoint rooted for legibility. Node support values <1 are denoted. The data underlying this figure can be found in https://doi.org/10.26188/21892974.v1. (TIF) [file pbio.3001879.s005.tif]

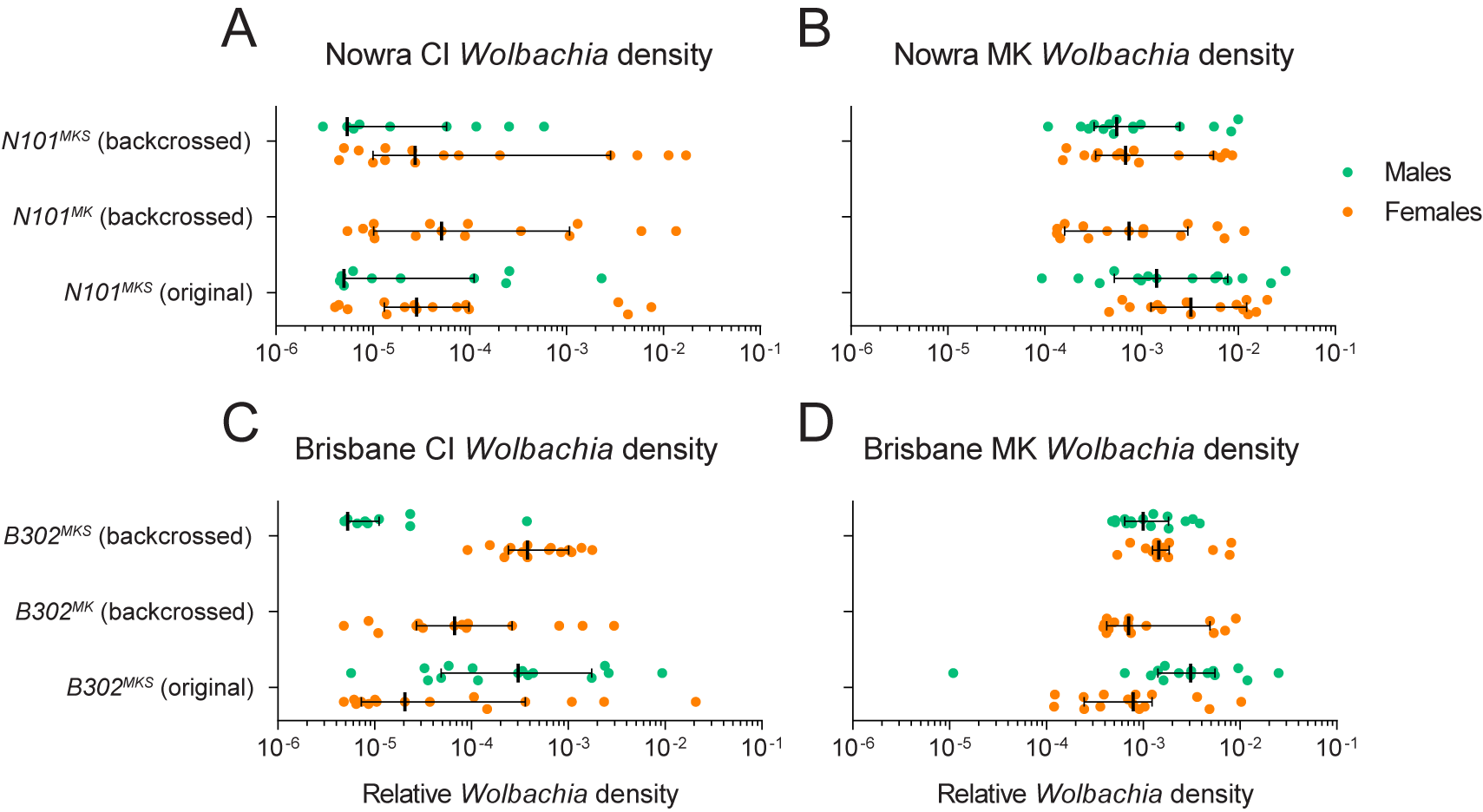

Supplement: S6 Fig — Females from the (A, B) N101MKS line or (C, D) B302MKS line were crossed to B116- males for 3 generations. Wolbachia density was measured in the original lines and backcrossed lines that produced both male and female offspring (MKS) or female-only offspring (MK). Data points show densities in individual adults, while vertical lines and error bars show medians and 95% confidence intervals. Individuals testing negative for a Wolbachia strain were excluded. The data underlying this figure can be found in https://doi.org/10.26188/21862119.v1. (TIF) [file pbio.3001879.s006.tif]
