# Supplementary material for: A male-killing Wolbachia endosymbiont is concealed by another endosymbiont and a nuclear suppressor
Source: PLoS Biol. 2023 Mar 22;21(3):e3001879. doi: 10.1371/journal.pbio.3001879 (PMC10069767; doi:10.1371/journal.pbio.3001879)
Supplement: S1 Table — (DOCX) [file pbio.3001879.s007.docx]

**S1 Table.** Collection locations for *D. pseudotakahashii* populations used in this study.

| **Collection area** | **Location** | | **# isofemale lines** | **Line names used in manuscript** |
| --- | --- | --- | --- | --- |
| **New South Wales** |  | | 37 |  |
| Nowra | 34.865°S, 150.534°E | | 34 | *N101^MK^, N51^CI^* |
| Moorland | 31.791°S, 152.651°E | | 3 |  |
|  |  | |  |  |
| **Southeastern Queensland** | |  | 128 |  |
| Mount Tamborine | 27.897°S, 153.180°E | | 38 | *B302^MK^* and *B305^MK^*, *B116^CI^*, *B302-*, *B305-, B116-* |
| " | 27.911°S, 153.187°E | | 19 |  |
| " | 27.929°S, 153.189°E | | 17 | *B256^MK^* |
| Cedar Creek | 27.862°S, 153.200°E | | 10 |  |
| Mount Glorious | 27.337°S, 152.770°E | | 44 | *B289^MK^*, *B246^MK^*, *B280^MK^* |
|  |  | |  |  |
| **Northern Queensland** |  | | 23 |  |
| Smithfield | 16.819°S, 145.684°E | | 5 | *Smith+* |
| Innisfail | 17.523°S, 146.028°E | | 3 |  |
| Cape Tribulation | 16.085°S, 145.454°E | | 1 |  |
| Lake Placid | 16.869°S, 145.674°E | | 3 |  |
| Townsville | 19.006°S, 146.212°E | | 9 | *Town3+*, *Town3-* |
| " | 18.211°S, 145.792°E | | 2 |  |
