# Supplementary material for: A male-killing Wolbachia endosymbiont is concealed by another endosymbiont and a nuclear suppressor
Source: PLoS Biol. 2023 Mar 22;21(3):e3001879. doi: 10.1371/journal.pbio.3001879 (PMC10069767; doi:10.1371/journal.pbio.3001879)
Supplement: S2 Table — (DOCX) [file pbio.3001879.s008.docx]

**S2 Table.** Infection types present in samples screened from lines originally expressing CI and MK phenotypes at F11

| **Line** | **Location** | **Phenotype** | **N per sex** | **Female % MK** | **Female % CI** | **Male % MK** | **Male % CI** |
| --- | --- | --- | --- | --- | --- | --- | --- |
| *B142* | Bris | CI | 2 | 0 | 100 | 0 | 50 |
| *B149* | Bris | CI | 2 | 0 | 100 | 0 | 50 |
| *B246* | Bris | MK | 2 | 100 | 100 | 100 | 50 |
| *B256* | Bris | MK | 2 | 50 | 100 | 100 | 50 |
| *B289* | Bris | MK | 2 | 100 | 100 | 100 | 0 |
| *B302* | Bris | MK | 2 | 100 | 100 | 100 | 100 |
| *B305* | Bris | MK | 2 | 100 | 100 | 100 | 50 |
| *N101* | Nowra | MK | 2 | 100 | 50 | 100 | 100 |
| *N40* | Nowra | CI | 2 | 0 | 100 | 0 | 0 |
