# Supplementary material for: A male-killing Wolbachia endosymbiont is concealed by another endosymbiont and a nuclear suppressor
Source: PLoS Biol. 2023 Mar 22;21(3):e3001879. doi: 10.1371/journal.pbio.3001879 (PMC10069767; doi:10.1371/journal.pbio.3001879)
Supplement: S3 Table — (DOCX) [file pbio.3001879.s009.docx]

**S3 Table.** Private allele counts, observed heterozygosity (H_O_), expected heterozygosity (H_E_), and inbreeding coefficients (F_IS_) for the female-only and mixed-sex phenotypes of the N101 and B302 lines.

| **Group** | **Private alleles** | **H_O_** | **H_E_** | **F_IS_** |
| --- | --- | --- | --- | --- |
| N101_Female | 471 | 0.00045 | 0.00040 | -0.00009 |
| N101_Mixed | 972 | 0.00054 | 0.00045 | -0.00021 |
| B302_Female | 432 | 0.00041 | 0.00036 | -0.00010 |
| B302_Mixed | 778 | 0.00057 | 0.00043 | -0.00029 |
