# Supplementary material for: A male-killing Wolbachia endosymbiont is concealed by another endosymbiont and a nuclear suppressor
Source: PLoS Biol. 2023 Mar 22;21(3):e3001879. doi: 10.1371/journal.pbio.3001879 (PMC10069767; doi:10.1371/journal.pbio.3001879)
Supplement: S4 Table — The swept region is on contig NW_025323476.1, starting at position 3,321,074 and ending at 4,637,826. The region is 1,316,752 bp in length. All 212 SNPs within this region have MAF of less than 0.1, whereas outside the region, higher-frequency SNPs are common. There are 131 unique genes within the region, with 153 unique gene products. (DOCX) [file pbio.3001879.s010.docx]

**S4 Table. Genes in region suspected of containing suppressor gene(s).** The swept region is on contig NW_025323476.1, starting at position 3,321,074 and ending at 4,637,826. The region is 1,316,752 bp in length. All 212 SNPs within this region have MAF of less than 0.1, whereas outside the region, higher-frequency SNPs are common. There are 131 unique genes within the region, with 153 unique gene products.

| contig | start | end | strand | exon | gene_id | product |
| --- | --- | --- | --- | --- | --- | --- |
| NW_025323476.1 | 3356781 | 3357113 | - | 0 | LOC108056003 | accessory gland protein Acp62F |
| NW_025323476.1 | 3358493 | 3358839 | - | 2 | LOC123002986 | accessory gland protein Acp62F-like |
| NW_025323476.1 | 3358957 | 3358994 | - | 1 | LOC123002986 | accessory gland protein Acp62F-like |
| NW_025323476.1 | 3359219 | 3359268 | - | 0 | LOC123002986 | accessory gland protein Acp62F-like |
| NW_025323476.1 | 3359272 | 3359610 | - | 0 | LOC108056007 | accessory gland protein Acp62F-like |
| NW_025323476.1 | 3360118 | 3360462 | - | 0 | LOC108056005 | accessory gland protein Acp62F-like |
| NW_025323476.1 | 3436739 | 3441280 | - | 0 | LOC108055249 | toll-like receptor 6 |
| NW_025323476.1 | 3471026 | 3472437 | - | 2 | LOC108055247 | cationic amino acid transporter 3 |
| NW_025323476.1 | 3472527 | 3472802 | - | 2 | LOC108055247 | cationic amino acid transporter 3 |
| NW_025323476.1 | 3472864 | 3473679 | - | 2 | LOC108055247 | cationic amino acid transporter 3 |
| NW_025323476.1 | 3473856 | 3473960 | - | 2 | LOC108055247 | cationic amino acid transporter 3 |
| NW_025323476.1 | 3474018 | 3474302 | - | 2 | LOC108055247 | cationic amino acid transporter 3 |
| NW_025323476.1 | 3474495 | 3474735 | - | 0 | LOC108055247 | cationic amino acid transporter 3 |
| NW_025323476.1 | 3481959 | 3482029 | + | 0 | LOC108055226 | bestrophin-4 |
| NW_025323476.1 | 3482091 | 3482201 | + | 1 | LOC108055226 | bestrophin-4 |
| NW_025323476.1 | 3482469 | 3483012 | + | 1 | LOC108055226 | bestrophin-4 |
| NW_025323476.1 | 3483066 | 3483944 | + | 0 | LOC108055226 | bestrophin-4 |
| NW_025323476.1 | 3484173 | 3484411 | - | 2 | LOC108055227 | bestrophin-4 |
| NW_025323476.1 | 3484481 | 3484712 | - | 0 | LOC108055227 | bestrophin-4 |
| NW_025323476.1 | 3484811 | 3485008 | - | 0 | LOC108055227 | bestrophin-4 |
| NW_025323476.1 | 3485139 | 3485886 | - | 1 | LOC108055227 | bestrophin-4 |
| NW_025323476.1 | 3485937 | 3486047 | - | 1 | LOC108055227 | bestrophin-4 |
| NW_025323476.1 | 3486106 | 3486176 | - | 0 | LOC108055227 | bestrophin-4 |
| NW_025323476.1 | 3538112 | 3542164 | - | 0 | LOC108055246 | toll-like receptor Tollo |
| NW_025323476.1 | 3573318 | 3573905 | - | 0 | LOC108055230 | peroxiredoxin 1 |
| NW_025323476.1 | 3621958 | 3622045 | + | 0 | LOC108055258 | endoplasmic reticulum-Golgi intermediate compartment protein 3 |
| NW_025323476.1 | 3622114 | 3622465 | + | 2 | LOC108055258 | endoplasmic reticulum-Golgi intermediate compartment protein 3 |
| NW_025323476.1 | 3623380 | 3623717 | + | 1 | LOC108055258 | endoplasmic reticulum-Golgi intermediate compartment protein 3 |
| NW_025323476.1 | 3623865 | 3623970 | - | 1 | LOC108055259 | reactive oxygen species modulator 1 |
| NW_025323476.1 | 3624086 | 3624216 | - | 0 | LOC108055259 | reactive oxygen species modulator 1 |
| NW_025323476.1 | 3624880 | 3625087 | + | 2 | LOC108055258 | endoplasmic reticulum-Golgi intermediate compartment protein 3 |
| NW_025323476.1 | 3625149 | 3625281 | + | 1 | LOC108055258 | endoplasmic reticulum-Golgi intermediate compartment protein 3 |
| NW_025323476.1 | 3625411 | 3625743 | - | 0 | LOC108055257 | U4/U6 small nuclear ribonucleoprotein Prp31 |
| NW_025323476.1 | 3625803 | 3626421 | - | 1 | LOC108055257 | U4/U6 small nuclear ribonucleoprotein Prp31 |
| NW_025323476.1 | 3626484 | 3626935 | - | 0 | LOC108055257 | U4/U6 small nuclear ribonucleoprotein Prp31 |
| NW_025323476.1 | 3627001 | 3627099 | - | 0 | LOC108055257 | U4/U6 small nuclear ribonucleoprotein Prp31 |
| NW_025323476.1 | 3627354 | 3628346 | + | 0 | LOC108055235 | probable DNA mismatch repair protein Msh6 |
| NW_025323476.1 | 3629042 | 3629371 | + | 0 | LOC108055235 | probable DNA mismatch repair protein Msh6 |
| NW_025323476.1 | 3629429 | 3631036 | + | 0 | LOC108055235 | probable DNA mismatch repair protein Msh6 |
| NW_025323476.1 | 3631847 | 3632332 | + | 0 | LOC108055235 | probable DNA mismatch repair protein Msh6 |
| NW_025323476.1 | 3632391 | 3632534 | + | 0 | LOC108055235 | probable DNA mismatch repair protein Msh6 |
| NW_025323476.1 | 3634078 | 3634599 | - | 0 | LOC108055236 | glycoprotein 3-alpha-L-fucosyltransferase A |
| NW_025323476.1 | 3635026 | 3635209 | - | 1 | LOC108055236 | glycoprotein 3-alpha-L-fucosyltransferase A |
| NW_025323476.1 | 3635495 | 3636214 | - | 1 | LOC108055236 | glycoprotein 3-alpha-L-fucosyltransferase A |
| NW_025323476.1 | 3636561 | 3636646 | - | 0 | LOC108055236 | glycoprotein 3-alpha-L-fucosyltransferase A |
| NW_025323476.1 | 3644100 | 3645251 | - | 0 | LOC108055271 | peroxisomal biogenesis factor 3 |
| NW_025323476.1 | 3646559 | 3646771 | + | 0 | LOC108055229 | pyroglutamyl-peptidase 1 |
| NW_025323476.1 | 3646845 | 3647288 | + | 0 | LOC108055229 | pyroglutamyl-peptidase 1 |
| NW_025323476.1 | 3648975 | 3649128 | + | 0 | LOC108055254 | protein disulfide-isomerase |
| NW_025323476.1 | 3652035 | 3653368 | + | 2 | LOC108055254 | protein disulfide-isomerase |
| NW_025323476.1 | 3655056 | 3655336 | + | 0 | LOC108055263 | uncharacterized protein LOC108055263 |
| NW_025323476.1 | 3655406 | 3655771 | + | 1 | LOC108055263 | uncharacterized protein LOC108055263 |
| NW_025323476.1 | 3656524 | 3656854 | + | 1 | LOC108055263 | uncharacterized protein LOC108055263 |
| NW_025323476.1 | 3657407 | 3658199 | + | 0 | LOC108055263 | uncharacterized protein LOC108055263 |
| NW_025323476.1 | 3658474 | 3660077 | + | 2 | LOC108055263 | uncharacterized protein LOC108055263 |
| NW_025323476.1 | 3660599 | 3660620 | + | 0 | LOC108055237 | uncharacterized protein LOC108055237 |
| NW_025323476.1 | 3660675 | 3663713 | + | 2 | LOC108055237 | uncharacterized protein LOC108055237 |
| NW_025323476.1 | 3664062 | 3664249 | + | 2 | LOC108055237 | uncharacterized protein LOC108055237 |
| NW_025323476.1 | 3664380 | 3664538 | - | 0 | LOC108055238 | centrosomal protein of 135 kDa isoform X2 |
| NW_025323476.1 | 3664380 | 3664538 | - | 0 | LOC108055238 | centrosomal protein of 135 kDa isoform X1 |
| NW_025323476.1 | 3664739 | 3664924 | - | 0 | LOC108055238 | centrosomal protein of 135 kDa isoform X2 |
| NW_025323476.1 | 3664739 | 3664924 | - | 0 | LOC108055238 | centrosomal protein of 135 kDa isoform X1 |
| NW_025323476.1 | 3665107 | 3665374 | - | 1 | LOC108055238 | centrosomal protein of 135 kDa isoform X2 |
| NW_025323476.1 | 3665107 | 3665374 | - | 1 | LOC108055238 | centrosomal protein of 135 kDa isoform X1 |
| NW_025323476.1 | 3666082 | 3666188 | - | 0 | LOC108055238 | centrosomal protein of 135 kDa isoform X2 |
| NW_025323476.1 | 3666082 | 3666206 | - | 0 | LOC108055238 | centrosomal protein of 135 kDa isoform X1 |
| NW_025323476.1 | 3666262 | 3666415 | - | 1 | LOC108055238 | centrosomal protein of 135 kDa isoform X2 |
| NW_025323476.1 | 3666262 | 3666415 | - | 1 | LOC108055238 | centrosomal protein of 135 kDa isoform X1 |
| NW_025323476.1 | 3666533 | 3666658 | - | 1 | LOC108055238 | centrosomal protein of 135 kDa isoform X2 |
| NW_025323476.1 | 3666533 | 3666658 | - | 1 | LOC108055238 | centrosomal protein of 135 kDa isoform X1 |
| NW_025323476.1 | 3672863 | 3673510 | - | 0 | LOC108055242 | E3 ubiquitin-protein ligase RNF185 |
| NW_025323476.1 | 3676368 | 3676483 | - | 0 | LOC108055238 | centrosomal protein of 135 kDa isoform X2 |
| NW_025323476.1 | 3676368 | 3676483 | - | 0 | LOC108055238 | centrosomal protein of 135 kDa isoform X1 |
| NW_025323476.1 | 3676544 | 3676754 | - | 1 | LOC108055238 | centrosomal protein of 135 kDa isoform X2 |
| NW_025323476.1 | 3676544 | 3676754 | - | 1 | LOC108055238 | centrosomal protein of 135 kDa isoform X1 |
| NW_025323476.1 | 3676818 | 3678213 | - | 2 | LOC108055238 | centrosomal protein of 135 kDa isoform X2 |
| NW_025323476.1 | 3676818 | 3678213 | - | 2 | LOC108055238 | centrosomal protein of 135 kDa isoform X1 |
| NW_025323476.1 | 3678271 | 3678423 | - | 2 | LOC108055238 | centrosomal protein of 135 kDa isoform X2 |
| NW_025323476.1 | 3678271 | 3678423 | - | 2 | LOC108055238 | centrosomal protein of 135 kDa isoform X1 |
| NW_025323476.1 | 3678483 | 3678609 | - | 0 | LOC108055238 | centrosomal protein of 135 kDa isoform X2 |
| NW_025323476.1 | 3678483 | 3678609 | - | 0 | LOC108055238 | centrosomal protein of 135 kDa isoform X1 |
| NW_025323476.1 | 3679061 | 3679135 | - | 0 | LOC108055238 | centrosomal protein of 135 kDa isoform X2 |
| NW_025323476.1 | 3679061 | 3679135 | - | 0 | LOC108055238 | centrosomal protein of 135 kDa isoform X1 |
| NW_025323476.1 | 3679200 | 3679298 | - | 0 | LOC108055238 | centrosomal protein of 135 kDa isoform X2 |
| NW_025323476.1 | 3679200 | 3679298 | - | 0 | LOC108055238 | centrosomal protein of 135 kDa isoform X1 |
| NW_025323476.1 | 3680381 | 3680632 | - | 0 | LOC108055260 | clavesin-2 |
| NW_025323476.1 | 3681643 | 3681933 | - | 0 | LOC108055260 | clavesin-2 |
| NW_025323476.1 | 3682724 | 3683181 | - | 2 | LOC108055260 | clavesin-2 |
| NW_025323476.1 | 3683793 | 3683802 | - | 0 | LOC108055260 | clavesin-2 |
| NW_025323476.1 | 3688725 | 3688919 | - | 0 | LOC108055251 | 1,5-anhydro-D-fructose reductase isoform X1 |
| NW_025323476.1 | 3688977 | 3689492 | - | 0 | LOC108055251 | 1,5-anhydro-D-fructose reductase isoform X1 |
| NW_025323476.1 | 3689548 | 3689715 | - | 0 | LOC108055251 | 1,5-anhydro-D-fructose reductase isoform X1 |
| NW_025323476.1 | 3690530 | 3690706 | - | 0 | LOC108055251 | aldo-keto reductase family 1 member B1 isoform X2 |
| NW_025323476.1 | 3690776 | 3691118 | - | 1 | LOC108055251 | aldo-keto reductase family 1 member B1 isoform X2 |
| NW_025323476.1 | 3691173 | 3691342 | - | 0 | LOC108055251 | aldo-keto reductase family 1 member B1 isoform X2 |
| NW_025323476.1 | 3691404 | 3691571 | - | 0 | LOC108055251 | aldo-keto reductase family 1 member B1 isoform X2 |
| NW_025323476.1 | 3691702 | 3691773 | - | 0 | LOC108055251 | 1,5-anhydro-D-fructose reductase isoform X1 |
| NW_025323476.1 | 3691702 | 3691773 | - | 0 | LOC108055251 | aldo-keto reductase family 1 member B1 isoform X2 |
| NW_025323476.1 | 3692302 | 3692468 | + | 0 | LOC108055252 | kxDL motif-containing protein CG10681 |
| NW_025323476.1 | 3692526 | 3693009 | + | 1 | LOC108055252 | kxDL motif-containing protein CG10681 |
| NW_025323476.1 | 3693692 | 3694675 | - | 0 | LOC108055250 | EARP-interacting protein homolog |
| NW_025323476.1 | 3694727 | 3694897 | - | 0 | LOC108055250 | EARP-interacting protein homolog |
| NW_025323476.1 | 3695461 | 3695567 | + | 0 | LOC108055268 | ubiquitin-conjugating enzyme E2 C |
| NW_025323476.1 | 3695631 | 3696057 | + | 1 | LOC108055268 | ubiquitin-conjugating enzyme E2 C |
| NW_025323476.1 | 3699924 | 3701527 | - | 2 | LOC108055232 | citron Rho-interacting kinase |
| NW_025323476.1 | 3701596 | 3701791 | - | 0 | LOC108055232 | citron Rho-interacting kinase |
| NW_025323476.1 | 3701849 | 3703351 | - | 0 | LOC108055232 | citron Rho-interacting kinase |
| NW_025323476.1 | 3704277 | 3705644 | - | 0 | LOC108055232 | citron Rho-interacting kinase |
| NW_025323476.1 | 3705716 | 3706066 | - | 0 | LOC108055232 | citron Rho-interacting kinase |
| NW_025323476.1 | 3706125 | 3706369 | - | 2 | LOC108055232 | citron Rho-interacting kinase |
| NW_025323476.1 | 3706849 | 3707122 | - | 0 | LOC108055232 | citron Rho-interacting kinase |
| NW_025323476.1 | 3707914 | 3708034 | + | 0 | LOC108055234 | protein LSM14 homolog B-A isoform X1 |
| NW_025323476.1 | 3707914 | 3708034 | + | 0 | LOC108055234 | protein LSM14 homolog B-A isoform X5 |
| NW_025323476.1 | 3707914 | 3708034 | + | 0 | LOC108055234 | protein LSM14 homolog B-A isoform X3 |
| NW_025323476.1 | 3707914 | 3708034 | + | 0 | LOC108055234 | protein LSM14 homolog B-A isoform X2 |
| NW_025323476.1 | 3707914 | 3708034 | + | 0 | LOC108055234 | protein LSM14 homolog B-A isoform X6 |
| NW_025323476.1 | 3707914 | 3708034 | + | 0 | LOC108055234 | protein LSM14 homolog B-A isoform X4 |
| NW_025323476.1 | 3707914 | 3708034 | + | 0 | LOC108055234 | protein LSM14 homolog B-A isoform X7 |
| NW_025323476.1 | 3709069 | 3709572 | + | 2 | LOC108055234 | protein LSM14 homolog B-A isoform X1 |
| NW_025323476.1 | 3709069 | 3709572 | + | 2 | LOC108055234 | protein LSM14 homolog B-A isoform X5 |
| NW_025323476.1 | 3709069 | 3709572 | + | 2 | LOC108055234 | protein LSM14 homolog B-A isoform X3 |
| NW_025323476.1 | 3709069 | 3709572 | + | 2 | LOC108055234 | protein LSM14 homolog B-A isoform X2 |
| NW_025323476.1 | 3709069 | 3709572 | + | 2 | LOC108055234 | protein LSM14 homolog B-A isoform X6 |
| NW_025323476.1 | 3709069 | 3709572 | + | 2 | LOC108055234 | protein LSM14 homolog B-A isoform X4 |
| NW_025323476.1 | 3709069 | 3709572 | + | 2 | LOC108055234 | protein LSM14 homolog B-A isoform X7 |
| NW_025323476.1 | 3710310 | 3710866 | + | 2 | LOC108055234 | protein LSM14 homolog B-A isoform X1 |
| NW_025323476.1 | 3710310 | 3710866 | + | 2 | LOC108055234 | protein LSM14 homolog B-A isoform X5 |
| NW_025323476.1 | 3710310 | 3710866 | + | 2 | LOC108055234 | protein LSM14 homolog B-A isoform X2 |
| NW_025323476.1 | 3710310 | 3710866 | + | 2 | LOC108055234 | protein LSM14 homolog B-A isoform X6 |
| NW_025323476.1 | 3710322 | 3710866 | + | 2 | LOC108055234 | protein LSM14 homolog B-A isoform X3 |
| NW_025323476.1 | 3710322 | 3710866 | + | 2 | LOC108055234 | protein LSM14 homolog B-A isoform X4 |
| NW_025323476.1 | 3710322 | 3710866 | + | 2 | LOC108055234 | protein LSM14 homolog B-A isoform X7 |
| NW_025323476.1 | 3710934 | 3711131 | + | 0 | LOC108055234 | protein LSM14 homolog B-A isoform X5 |
| NW_025323476.1 | 3710934 | 3711131 | + | 0 | LOC108055234 | protein LSM14 homolog B-A isoform X6 |
| NW_025323476.1 | 3710934 | 3711131 | + | 0 | LOC108055234 | protein LSM14 homolog B-A isoform X7 |
| NW_025323476.1 | 3710934 | 3711161 | + | 0 | LOC108055234 | protein LSM14 homolog B-A isoform X1 |
| NW_025323476.1 | 3710934 | 3711161 | + | 0 | LOC108055234 | protein LSM14 homolog B-A isoform X3 |
| NW_025323476.1 | 3710934 | 3711161 | + | 0 | LOC108055234 | protein LSM14 homolog B-A isoform X2 |
| NW_025323476.1 | 3710934 | 3711161 | + | 0 | LOC108055234 | protein LSM14 homolog B-A isoform X4 |
| NW_025323476.1 | 3712117 | 3712346 | + | 0 | LOC108055234 | protein LSM14 homolog B-A isoform X1 |
| NW_025323476.1 | 3712117 | 3712346 | + | 0 | LOC108055234 | protein LSM14 homolog B-A isoform X5 |
| NW_025323476.1 | 3712117 | 3712346 | + | 0 | LOC108055234 | protein LSM14 homolog B-A isoform X3 |
| NW_025323476.1 | 3712117 | 3712346 | + | 0 | LOC108055234 | protein LSM14 homolog B-A isoform X2 |
| NW_025323476.1 | 3712117 | 3712346 | + | 0 | LOC108055234 | protein LSM14 homolog B-A isoform X6 |
| NW_025323476.1 | 3712117 | 3712346 | + | 0 | LOC108055234 | protein LSM14 homolog B-A isoform X4 |
| NW_025323476.1 | 3712117 | 3712346 | + | 0 | LOC108055234 | protein LSM14 homolog B-A isoform X7 |
| NW_025323476.1 | 3712409 | 3712758 | + | 1 | LOC108055234 | protein LSM14 homolog B-A isoform X1 |
| NW_025323476.1 | 3712409 | 3712758 | + | 1 | LOC108055234 | protein LSM14 homolog B-A isoform X5 |
| NW_025323476.1 | 3712409 | 3712758 | + | 1 | LOC108055234 | protein LSM14 homolog B-A isoform X3 |
| NW_025323476.1 | 3712409 | 3712763 | + | 1 | LOC108055234 | protein LSM14 homolog B-A isoform X2 |
| NW_025323476.1 | 3712409 | 3712763 | + | 1 | LOC108055234 | protein LSM14 homolog B-A isoform X6 |
| NW_025323476.1 | 3712409 | 3712763 | + | 1 | LOC108055234 | protein LSM14 homolog B-A isoform X4 |
| NW_025323476.1 | 3712409 | 3712763 | + | 1 | LOC108055234 | protein LSM14 homolog B-A isoform X7 |
| NW_025323476.1 | 3712828 | 3712832 | + | 2 | LOC108055234 | protein LSM14 homolog B-A isoform X1 |
| NW_025323476.1 | 3712828 | 3712832 | + | 2 | LOC108055234 | protein LSM14 homolog B-A isoform X5 |
| NW_025323476.1 | 3712828 | 3712832 | + | 2 | LOC108055234 | protein LSM14 homolog B-A isoform X3 |
| NW_025323476.1 | 3714959 | 3715485 | - | 2 | LOC108055233 | histone acetyltransferase KAT2A |
| NW_025323476.1 | 3715548 | 3716200 | - | 1 | LOC108055233 | histone acetyltransferase KAT2A |
| NW_025323476.1 | 3716252 | 3717513 | - | 0 | LOC108055233 | histone acetyltransferase KAT2A |
| NW_025323476.1 | 3718182 | 3718826 | + | 0 | LOC108055273 | uncharacterized protein LOC108055273 |
| NW_025323476.1 | 3718903 | 3719423 | - | 2 | LOC108055272 | eukaryotic translation initiation factor 2 subunit 2 |
| NW_025323476.1 | 3719988 | 3720384 | - | 0 | LOC108055272 | eukaryotic translation initiation factor 2 subunit 2 |
| NW_025323476.1 | 3720579 | 3720593 | - | 0 | LOC108055272 | eukaryotic translation initiation factor 2 subunit 2 |
| NW_025323476.1 | 3722061 | 3722156 | - | 0 | LOC108055270 | phosphoacetylglucosamine mutase |
| NW_025323476.1 | 3722219 | 3723662 | - | 1 | LOC108055270 | phosphoacetylglucosamine mutase |
| NW_025323476.1 | 3724680 | 3724780 | - | 0 | LOC108055270 | phosphoacetylglucosamine mutase |
| NW_025323476.1 | 3725674 | 3726729 | - | 0 | LOC108055262 | melanotransferrin |
| NW_025323476.1 | 3727142 | 3727306 | - | 0 | LOC108055262 | melanotransferrin |
| NW_025323476.1 | 3727367 | 3727818 | - | 2 | LOC108055262 | melanotransferrin |
| NW_025323476.1 | 3727878 | 3728038 | - | 1 | LOC108055262 | melanotransferrin |
| NW_025323476.1 | 3728103 | 3728236 | - | 0 | LOC108055262 | melanotransferrin |
| NW_025323476.1 | 3728720 | 3729147 | - | 2 | LOC108055262 | melanotransferrin |
| NW_025323476.1 | 3729227 | 3729287 | - | 0 | LOC108055262 | melanotransferrin |
| NW_025323476.1 | 3729852 | 3730025 | + | 0 | LOC108055231 | uncharacterized protein LOC108055231 |
| NW_025323476.1 | 3730598 | 3731359 | + | 0 | LOC108055265 | phosphomannomutase |
| NW_025323476.1 | 3731460 | 3732142 | - | 2 | LOC108055264 | kelch domain-containing protein 4 |
| NW_025323476.1 | 3732203 | 3732623 | - | 0 | LOC108055264 | kelch domain-containing protein 4 |
| NW_025323476.1 | 3732890 | 3733195 | - | 0 | LOC108055264 | kelch domain-containing protein 4 |
| NW_025323476.1 | 3733254 | 3733367 | - | 0 | LOC108055264 | kelch domain-containing protein 4 |
| NW_025323476.1 | 3733961 | 3734643 | - | 2 | LOC108055243 | uncharacterized protein LOC108055243 |
| NW_025323476.1 | 3734760 | 3734786 | - | 2 | LOC108055243 | uncharacterized protein LOC108055243 |
| NW_025323476.1 | 3736255 | 3736327 | - | 0 | LOC108055243 | uncharacterized protein LOC108055243 |
| NW_025323476.1 | 3746401 | 3746421 | - | 0 | LOC108055244 | ankyrin repeat domain-containing protein SOWAHB-like |
| NW_025323476.1 | 3764696 | 3764836 | - | 0 | LOC108055244 | ankyrin repeat domain-containing protein SOWAHB-like |
| NW_025323476.1 | 3772269 | 3772915 | - | 2 | LOC108055244 | ankyrin repeat domain-containing protein SOWAHB-like |
| NW_025323476.1 | 3773009 | 3773135 | - | 0 | LOC108055244 | ankyrin repeat domain-containing protein SOWAHB-like |
| NW_025323476.1 | 3784245 | 3784295 | + | 0 | LOC108055269 | homeobox protein araucan isoform X1 |
| NW_025323476.1 | 3784245 | 3784295 | + | 0 | LOC108055269 | homeobox protein araucan isoform X2 |
| NW_025323476.1 | 3788895 | 3789449 | + | 0 | LOC108055269 | homeobox protein araucan isoform X1 |
| NW_025323476.1 | 3788895 | 3789449 | + | 0 | LOC108055269 | homeobox protein araucan isoform X2 |
| NW_025323476.1 | 3795664 | 3795779 | + | 0 | LOC108055269 | homeobox protein araucan isoform X1 |
| NW_025323476.1 | 3795664 | 3795779 | + | 0 | LOC108055269 | homeobox protein araucan isoform X2 |
| NW_025323476.1 | 3800531 | 3800886 | + | 1 | LOC108055269 | homeobox protein araucan isoform X1 |
| NW_025323476.1 | 3800555 | 3800886 | + | 1 | LOC108055269 | homeobox protein araucan isoform X2 |
| NW_025323476.1 | 3800955 | 3802033 | + | 2 | LOC108055269 | homeobox protein araucan isoform X1 |
| NW_025323476.1 | 3800955 | 3802033 | + | 2 | LOC108055269 | homeobox protein araucan isoform X2 |
| NW_025323476.1 | 3820017 | 3820067 | + | 0 | LOC108061641 | homeobox protein caupolican isoform X1 |
| NW_025323476.1 | 3820017 | 3820067 | + | 0 | LOC108061641 | homeobox protein caupolican isoform X2 |
| NW_025323476.1 | 3826148 | 3826630 | + | 0 | LOC108061641 | homeobox protein caupolican isoform X1 |
| NW_025323476.1 | 3826148 | 3826630 | + | 0 | LOC108061641 | homeobox protein caupolican isoform X2 |
| NW_025323476.1 | 3826874 | 3826992 | + | 0 | LOC108061641 | homeobox protein caupolican isoform X1 |
| NW_025323476.1 | 3826874 | 3826992 | + | 0 | LOC108061641 | homeobox protein caupolican isoform X2 |
| NW_025323476.1 | 3827920 | 3828242 | + | 1 | LOC108061641 | homeobox protein caupolican isoform X1 |
| NW_025323476.1 | 3827950 | 3828242 | + | 1 | LOC108061641 | homeobox protein caupolican isoform X2 |
| NW_025323476.1 | 3829398 | 3830416 | + | 2 | LOC108061641 | homeobox protein caupolican isoform X1 |
| NW_025323476.1 | 3829398 | 3830416 | + | 2 | LOC108061641 | homeobox protein caupolican isoform X2 |
| NW_025323476.1 | 3857498 | 3857562 | + | 0 | LOC123002987 | uncharacterized protein LOC123002987 |
| NW_025323476.1 | 3857627 | 3857670 | + | 1 | LOC123002987 | uncharacterized protein LOC123002987 |
| NW_025323476.1 | 3857737 | 3857818 | + | 2 | LOC123002987 | uncharacterized protein LOC123002987 |
| NW_025323476.1 | 3862349 | 3862457 | + | 1 | LOC123002987 | uncharacterized protein LOC123002987 |
| NW_025323476.1 | 3915221 | 3915262 | + | 0 | LOC108061723 | homeobox protein caupolican |
| NW_025323476.1 | 3917515 | 3918018 | + | 0 | LOC108061723 | homeobox protein caupolican |
| NW_025323476.1 | 3926320 | 3926426 | + | 0 | LOC108061723 | homeobox protein caupolican |
| NW_025323476.1 | 3928279 | 3928577 | + | 1 | LOC108061723 | homeobox protein caupolican |
| NW_025323476.1 | 3929219 | 3930201 | + | 2 | LOC108061723 | homeobox protein caupolican |
| NW_025323476.1 | 3960489 | 3960846 | - | 1 | LOC108061660 | probable small nuclear ribonucleoprotein Sm D1 |
| NW_025323476.1 | 3961349 | 3961362 | - | 0 | LOC108061660 | probable small nuclear ribonucleoprotein Sm D1 |
| NW_025323476.1 | 3962309 | 3962405 | + | 0 | LOC108061721 | tyrosine-protein phosphatase 69D |
| NW_025323476.1 | 3963184 | 3963286 | + | 2 | LOC108061721 | tyrosine-protein phosphatase 69D |
| NW_025323476.1 | 3963347 | 3965190 | + | 1 | LOC108061721 | tyrosine-protein phosphatase 69D |
| NW_025323476.1 | 3965252 | 3965497 | + | 2 | LOC108061721 | tyrosine-protein phosphatase 69D |
| NW_025323476.1 | 3965556 | 3965799 | + | 2 | LOC108061721 | tyrosine-protein phosphatase 69D |
| NW_025323476.1 | 3965870 | 3966758 | + | 1 | LOC108061721 | tyrosine-protein phosphatase 69D |
| NW_025323476.1 | 3966818 | 3966964 | + | 0 | LOC108061721 | tyrosine-protein phosphatase 69D |
| NW_025323476.1 | 3967021 | 3967131 | + | 0 | LOC108061721 | tyrosine-protein phosphatase 69D |
| NW_025323476.1 | 3967193 | 3967372 | + | 0 | LOC108061721 | tyrosine-protein phosphatase 69D |
| NW_025323476.1 | 3967429 | 3967736 | + | 0 | LOC108061721 | tyrosine-protein phosphatase 69D |
| NW_025323476.1 | 3967795 | 3967930 | + | 1 | LOC108061721 | tyrosine-protein phosphatase 69D |
| NW_025323476.1 | 3967993 | 3968067 | + | 0 | LOC108061721 | tyrosine-protein phosphatase 69D |
| NW_025323476.1 | 3968913 | 3968957 | + | 0 | LOC123002978 | tyrosine-protein phosphatase 69D-like |
| NW_025323476.1 | 3969014 | 3969321 | + | 0 | LOC123002978 | tyrosine-protein phosphatase 69D-like |
| NW_025323476.1 | 3969380 | 3969515 | + | 1 | LOC123002978 | tyrosine-protein phosphatase 69D-like |
| NW_025323476.1 | 3969578 | 3969652 | + | 0 | LOC123002978 | tyrosine-protein phosphatase 69D-like |
| NW_025323476.1 | 3970591 | 3970682 | + | 0 | LOC108061698 | protein ABHD18 |
| NW_025323476.1 | 3971645 | 3971912 | + | 1 | LOC108061698 | protein ABHD18 |
| NW_025323476.1 | 3972303 | 3972415 | + | 0 | LOC108061698 | protein ABHD18 |
| NW_025323476.1 | 3972479 | 3972617 | + | 1 | LOC108061698 | protein ABHD18 |
| NW_025323476.1 | 3972677 | 3972766 | + | 0 | LOC108061698 | protein ABHD18 |
| NW_025323476.1 | 3972822 | 3973531 | + | 0 | LOC108061698 | protein ABHD18 |
| NW_025323476.1 | 3973590 | 3973710 | + | 1 | LOC108061698 | protein ABHD18 |
| NW_025323476.1 | 3974039 | 3974099 | + | 0 | LOC108061700 | uncharacterized protein LOC108061700 |
| NW_025323476.1 | 3974399 | 3974595 | + | 2 | LOC108061700 | uncharacterized protein LOC108061700 |
| NW_025323476.1 | 3974660 | 3974918 | + | 0 | LOC108061700 | uncharacterized protein LOC108061700 |
| NW_025323476.1 | 3974979 | 3975157 | + | 2 | LOC108061700 | uncharacterized protein LOC108061700 |
| NW_025323476.1 | 3975487 | 3975501 | - | 0 | LOC108061697 | kinesin light chain isoform X2 |
| NW_025323476.1 | 3975487 | 3975501 | - | 0 | LOC108061697 | kinesin light chain isoform X2 |
| NW_025323476.1 | 3975487 | 3975501 | - | 0 | LOC108061697 | kinesin light chain isoform X1 |
| NW_025323476.1 | 3975561 | 3975866 | - | 0 | LOC108061697 | kinesin light chain isoform X2 |
| NW_025323476.1 | 3975561 | 3975866 | - | 0 | LOC108061697 | kinesin light chain isoform X2 |
| NW_025323476.1 | 3975561 | 3975866 | - | 0 | LOC108061697 | kinesin light chain isoform X1 |
| NW_025323476.1 | 3975936 | 3976415 | - | 0 | LOC108061697 | kinesin light chain isoform X2 |
| NW_025323476.1 | 3975936 | 3976415 | - | 0 | LOC108061697 | kinesin light chain isoform X2 |
| NW_025323476.1 | 3975936 | 3976415 | - | 0 | LOC108061697 | kinesin light chain isoform X1 |
| NW_025323476.1 | 3976475 | 3976595 | - | 1 | LOC108061697 | kinesin light chain isoform X2 |
| NW_025323476.1 | 3976475 | 3976595 | - | 1 | LOC108061697 | kinesin light chain isoform X2 |
| NW_025323476.1 | 3976475 | 3976595 | - | 1 | LOC108061697 | kinesin light chain isoform X1 |
| NW_025323476.1 | 3977324 | 3977925 | - | 0 | LOC108061697 | kinesin light chain isoform X2 |
| NW_025323476.1 | 3977324 | 3977925 | - | 0 | LOC108061697 | kinesin light chain isoform X2 |
| NW_025323476.1 | 3977324 | 3977950 | - | 1 | LOC108061697 | kinesin light chain isoform X1 |
| NW_025323476.1 | 3978076 | 3978110 | - | 0 | LOC108061697 | kinesin light chain isoform X1 |
| NW_025323476.1 | 3979939 | 3980068 | + | 0 | LOC108061695 | uncharacterized protein LOC108061695 |
| NW_025323476.1 | 3980514 | 3982129 | + | 2 | LOC108061695 | uncharacterized protein LOC108061695 |
| NW_025323476.1 | 3982190 | 3982360 | + | 0 | LOC108061695 | uncharacterized protein LOC108061695 |
| NW_025323476.1 | 3982511 | 3982558 | - | 0 | LOC108061699 | hsp70 nucleotide exchange factor fes1 |
| NW_025323476.1 | 3982617 | 3982825 | - | 2 | LOC108061699 | hsp70 nucleotide exchange factor fes1 |
| NW_025323476.1 | 3982893 | 3983436 | - | 0 | LOC108061699 | hsp70 nucleotide exchange factor fes1 |
| NW_025323476.1 | 3983991 | 3984068 | - | 0 | LOC108061699 | hsp70 nucleotide exchange factor fes1 |
| NW_025323476.1 | 3984126 | 3984167 | - | 0 | LOC108061699 | hsp70 nucleotide exchange factor fes1 |
| NW_025323476.1 | 3984560 | 3984656 | + | 0 | LOC108061701 | vacuolar protein sorting-associated protein 13D |
| NW_025323476.1 | 3984723 | 3985513 | + | 2 | LOC108061701 | vacuolar protein sorting-associated protein 13D |
| NW_025323476.1 | 3986159 | 3987205 | + | 0 | LOC108061701 | vacuolar protein sorting-associated protein 13D |
| NW_025323476.1 | 3987263 | 3988173 | + | 0 | LOC108061701 | vacuolar protein sorting-associated protein 13D |
| NW_025323476.1 | 3988408 | 3988538 | + | 1 | LOC108061701 | vacuolar protein sorting-associated protein 13D |
| NW_025323476.1 | 3988698 | 3988838 | + | 2 | LOC108061701 | vacuolar protein sorting-associated protein 13D |
| NW_025323476.1 | 3988900 | 3989524 | + | 2 | LOC108061701 | vacuolar protein sorting-associated protein 13D |
| NW_025323476.1 | 3989583 | 3989702 | + | 1 | LOC108061701 | vacuolar protein sorting-associated protein 13D |
| NW_025323476.1 | 3989763 | 3990087 | + | 1 | LOC108061701 | vacuolar protein sorting-associated protein 13D |
| NW_025323476.1 | 3990157 | 3990949 | + | 0 | LOC108061701 | vacuolar protein sorting-associated protein 13D |
| NW_025323476.1 | 3991003 | 3991255 | + | 2 | LOC108061701 | vacuolar protein sorting-associated protein 13D |
| NW_025323476.1 | 3991331 | 3991475 | + | 1 | LOC108061701 | vacuolar protein sorting-associated protein 13D |
| NW_025323476.1 | 3991538 | 3994870 | + | 0 | LOC108061701 | vacuolar protein sorting-associated protein 13D |
| NW_025323476.1 | 3994923 | 3995135 | + | 0 | LOC108061701 | vacuolar protein sorting-associated protein 13D |
| NW_025323476.1 | 3995201 | 3996055 | + | 0 | LOC108061701 | vacuolar protein sorting-associated protein 13D |
| NW_025323476.1 | 3996293 | 3996604 | + | 0 | LOC108061701 | vacuolar protein sorting-associated protein 13D |
| NW_025323476.1 | 3996662 | 3997168 | + | 0 | LOC108061701 | vacuolar protein sorting-associated protein 13D |
| NW_025323476.1 | 3997227 | 3998345 | + | 0 | LOC108061701 | vacuolar protein sorting-associated protein 13D |
| NW_025323476.1 | 3998566 | 3998613 | + | 0 | LOC108061701 | vacuolar protein sorting-associated protein 13D |
| NW_025323476.1 | 3998975 | 3999459 | - | 2 | LOC108061702 | huntingtin-interacting protein 1 |
| NW_025323476.1 | 3999516 | 3999920 | - | 2 | LOC108061702 | huntingtin-interacting protein 1 |
| NW_025323476.1 | 3999990 | 4001130 | - | 0 | LOC108061702 | huntingtin-interacting protein 1 |
| NW_025323476.1 | 4001184 | 4001293 | - | 2 | LOC108061702 | huntingtin-interacting protein 1 |
| NW_025323476.1 | 4001348 | 4002078 | - | 1 | LOC108061702 | huntingtin-interacting protein 1 |
| NW_025323476.1 | 4002137 | 4002483 | - | 0 | LOC108061702 | huntingtin-interacting protein 1 |
| NW_025323476.1 | 4009847 | 4010657 | - | 1 | LOC108061642 | uncharacterized protein LOC108061642 |
| NW_025323476.1 | 4011306 | 4011383 | - | 1 | LOC108061642 | uncharacterized protein LOC108061642 |
| NW_025323476.1 | 4011436 | 4011823 | - | 2 | LOC108061642 | uncharacterized protein LOC108061642 |
| NW_025323476.1 | 4012223 | 4012380 | - | 1 | LOC108061642 | uncharacterized protein LOC108061642 |
| NW_025323476.1 | 4012837 | 4013787 | - | 1 | LOC108061642 | uncharacterized protein LOC108061642 |
| NW_025323476.1 | 4013845 | 4013918 | - | 0 | LOC108061642 | uncharacterized protein LOC108061642 |
| NW_025323476.1 | 4014850 | 4016270 | - | 2 | LOC108061643 | uncharacterized protein LOC108061643 |
| NW_025323476.1 | 4016367 | 4017018 | - | 0 | LOC108061643 | uncharacterized protein LOC108061643 |
| NW_025323476.1 | 4017623 | 4017672 | + | 0 | LOC108061653 | uncharacterized protein LOC108061653 |
| NW_025323476.1 | 4017728 | 4019413 | + | 1 | LOC108061653 | uncharacterized protein LOC108061653 |
| NW_025323476.1 | 4019550 | 4019903 | + | 1 | LOC108061653 | uncharacterized protein LOC108061653 |
| NW_025323476.1 | 4020057 | 4020483 | + | 1 | LOC108061653 | uncharacterized protein LOC108061653 |
| NW_025323476.1 | 4020902 | 4021255 | - | 0 | LOC108061727 | endocuticle structural glycoprotein SgAbd-8 |
| NW_025323476.1 | 4021320 | 4021331 | - | 0 | LOC108061727 | endocuticle structural glycoprotein SgAbd-8 |
| NW_025323476.1 | 4022609 | 4022619 | + | 0 | LOC108061726 | MICOS complex subunit MIC13 homolog QIL1 |
| NW_025323476.1 | 4022690 | 4023044 | + | 1 | LOC108061726 | MICOS complex subunit MIC13 homolog QIL1 |
| NW_025323476.1 | 4028117 | 4028154 | + | 0 | LOC108061729 | pH-sensitive chloride channel 2 |
| NW_025323476.1 | 4031203 | 4031330 | + | 1 | LOC108061729 | pH-sensitive chloride channel 2 |
| NW_025323476.1 | 4031391 | 4031792 | + | 2 | LOC108061729 | pH-sensitive chloride channel 2 |
| NW_025323476.1 | 4032669 | 4032827 | + | 2 | LOC108061729 | pH-sensitive chloride channel 2 |
| NW_025323476.1 | 4032886 | 4033041 | + | 2 | LOC108061729 | pH-sensitive chloride channel 2 |
| NW_025323476.1 | 4033502 | 4033881 | + | 2 | LOC108061729 | pH-sensitive chloride channel 2 |
| NW_025323476.1 | 4033947 | 4034183 | + | 0 | LOC108061729 | pH-sensitive chloride channel 2 |
| NW_025323476.1 | 4035174 | 4035348 | + | 0 | LOC108061651 | cytochrome b-c1 complex subunit 8 |
| NW_025323476.1 | 4035407 | 4035498 | + | 2 | LOC108061651 | cytochrome b-c1 complex subunit 8 |
| NW_025323476.1 | 4036208 | 4036369 | + | 0 | LOC108061652 | uncharacterized protein LOC108061652 |
| NW_025323476.1 | 4037918 | 4038670 | - | 0 | LOC108061658 | uncharacterized protein LOC108061658 |
| NW_025323476.1 | 4039201 | 4039800 | - | 0 | LOC108061659 | uncharacterized protein LOC108061659 |
| NW_025323476.1 | 4055008 | 4055352 | + | 0 | LOC108061694 | uncharacterized protein LOC108061694 |
| NW_025323476.1 | 4069984 | 4071382 | + | 0 | LOC108061692 | solute carrier organic anion transporter family member 74D |
| NW_025323476.1 | 4069984 | 4071382 | + | 0 | LOC108061692 | solute carrier organic anion transporter family member 74D |
| NW_025323476.1 | 4072093 | 4072456 | + | 2 | LOC108061692 | solute carrier organic anion transporter family member 74D |
| NW_025323476.1 | 4072093 | 4072456 | + | 2 | LOC108061692 | solute carrier organic anion transporter family member 74D |
| NW_025323476.1 | 4076009 | 4076193 | + | 1 | LOC108061692 | solute carrier organic anion transporter family member 74D |
| NW_025323476.1 | 4076009 | 4076193 | + | 1 | LOC108061692 | solute carrier organic anion transporter family member 74D |
| NW_025323476.1 | 4076260 | 4076403 | + | 2 | LOC108061692 | solute carrier organic anion transporter family member 74D |
| NW_025323476.1 | 4076260 | 4076403 | + | 2 | LOC108061692 | solute carrier organic anion transporter family member 74D |
| NW_025323476.1 | 4076464 | 4076640 | + | 2 | LOC108061692 | solute carrier organic anion transporter family member 74D |
| NW_025323476.1 | 4076464 | 4076640 | + | 2 | LOC108061692 | solute carrier organic anion transporter family member 74D |
| NW_025323476.1 | 4077494 | 4077672 | + | 2 | LOC108061692 | solute carrier organic anion transporter family member 74D |
| NW_025323476.1 | 4077494 | 4077672 | + | 2 | LOC108061692 | solute carrier organic anion transporter family member 74D |
| NW_025323476.1 | 4078577 | 4080220 | - | 0 | LOC108061693 | U4/U6 small nuclear ribonucleoprotein Prp4 |
| NW_025323476.1 | 4081090 | 4082322 | + | 0 | LOC108061722 | putative RNA-binding protein Luc7-like 1 |
| NW_025323476.1 | 4086455 | 4086782 | - | 1 | LOC108061688 | enhancer of mRNA-decapping protein 3 |
| NW_025323476.1 | 4086839 | 4086923 | - | 2 | LOC108061688 | enhancer of mRNA-decapping protein 3 |
| NW_025323476.1 | 4086985 | 4087145 | - | 1 | LOC108061688 | enhancer of mRNA-decapping protein 3 |
| NW_025323476.1 | 4087207 | 4088127 | - | 1 | LOC108061688 | enhancer of mRNA-decapping protein 3 |
| NW_025323476.1 | 4088872 | 4089425 | - | 0 | LOC108061688 | enhancer of mRNA-decapping protein 3 |
| NW_025323476.1 | 4090751 | 4090942 | + | 0 | LOC108061686 | E3 ubiquitin-protein ligase Nedd-4 isoform X11 |
| NW_025323476.1 | 4090751 | 4090942 | + | 0 | LOC108061686 | E3 ubiquitin-protein ligase Nedd-4 isoform X13 |
| NW_025323476.1 | 4090751 | 4090942 | + | 0 | LOC108061686 | E3 ubiquitin-protein ligase Nedd-4 isoform X1 |
| NW_025323476.1 | 4090751 | 4090942 | + | 0 | LOC108061686 | E3 ubiquitin-protein ligase Nedd-4 isoform X3 |
| NW_025323476.1 | 4090751 | 4090942 | + | 0 | LOC108061686 | E3 ubiquitin-protein ligase Nedd-4 isoform X1 |
| NW_025323476.1 | 4090751 | 4090942 | + | 0 | LOC108061686 | E3 ubiquitin-protein ligase Nedd-4 isoform X4 |
| NW_025323476.1 | 4090751 | 4090942 | + | 0 | LOC108061686 | E3 ubiquitin-protein ligase Nedd-4 isoform X2 |
| NW_025323476.1 | 4090751 | 4090942 | + | 0 | LOC108061686 | E3 ubiquitin-protein ligase Nedd-4 isoform X5 |
| NW_025323476.1 | 4090751 | 4090942 | + | 0 | LOC108061686 | E3 ubiquitin-protein ligase Nedd-4 isoform X8 |
| NW_025323476.1 | 4090751 | 4090942 | + | 0 | LOC108061686 | E3 ubiquitin-protein ligase Nedd-4 isoform X9 |
| NW_025323476.1 | 4094595 | 4094669 | + | 0 | LOC108061686 | E3 ubiquitin-protein ligase Nedd-4 isoform X14 |
| NW_025323476.1 | 4094595 | 4094669 | + | 0 | LOC108061686 | E3 ubiquitin-protein ligase Nedd-4 isoform X7 |
| NW_025323476.1 | 4094595 | 4094669 | + | 0 | LOC108061686 | E3 ubiquitin-protein ligase Nedd-4 isoform X6 |
| NW_025323476.1 | 4094595 | 4094669 | + | 0 | LOC108061686 | E3 ubiquitin-protein ligase Nedd-4 isoform X10 |
| NW_025323476.1 | 4099637 | 4099719 | + | 0 | LOC108061686 | E3 ubiquitin-protein ligase Nedd-4 isoform X11 |
| NW_025323476.1 | 4099637 | 4099719 | + | 0 | LOC108061686 | E3 ubiquitin-protein ligase Nedd-4 isoform X13 |
| NW_025323476.1 | 4099637 | 4099719 | + | 0 | LOC108061686 | E3 ubiquitin-protein ligase Nedd-4 isoform X1 |
| NW_025323476.1 | 4099637 | 4099719 | + | 0 | LOC108061686 | E3 ubiquitin-protein ligase Nedd-4 isoform X3 |
| NW_025323476.1 | 4099637 | 4099719 | + | 0 | LOC108061686 | E3 ubiquitin-protein ligase Nedd-4 isoform X1 |
| NW_025323476.1 | 4099637 | 4099719 | + | 0 | LOC108061686 | E3 ubiquitin-protein ligase Nedd-4 isoform X4 |
| NW_025323476.1 | 4099637 | 4099719 | + | 0 | LOC108061686 | E3 ubiquitin-protein ligase Nedd-4 isoform X2 |
| NW_025323476.1 | 4099637 | 4099719 | + | 0 | LOC108061686 | E3 ubiquitin-protein ligase Nedd-4 isoform X5 |
| NW_025323476.1 | 4099637 | 4099719 | + | 0 | LOC108061686 | E3 ubiquitin-protein ligase Nedd-4 isoform X8 |
| NW_025323476.1 | 4099637 | 4099719 | + | 0 | LOC108061686 | E3 ubiquitin-protein ligase Nedd-4 isoform X9 |
| NW_025323476.1 | 4099637 | 4099719 | + | 0 | LOC108061686 | E3 ubiquitin-protein ligase Nedd-4 isoform X14 |
| NW_025323476.1 | 4099637 | 4099719 | + | 0 | LOC108061686 | E3 ubiquitin-protein ligase Nedd-4 isoform X7 |
| NW_025323476.1 | 4099637 | 4099719 | + | 0 | LOC108061686 | E3 ubiquitin-protein ligase Nedd-4 isoform X6 |
| NW_025323476.1 | 4099637 | 4099719 | + | 0 | LOC108061686 | E3 ubiquitin-protein ligase Nedd-4 isoform X10 |
| NW_025323476.1 | 4099789 | 4099870 | + | 1 | LOC108061686 | E3 ubiquitin-protein ligase Nedd-4 isoform X11 |
| NW_025323476.1 | 4099789 | 4099870 | + | 1 | LOC108061686 | E3 ubiquitin-protein ligase Nedd-4 isoform X13 |
| NW_025323476.1 | 4099789 | 4099870 | + | 1 | LOC108061686 | E3 ubiquitin-protein ligase Nedd-4 isoform X1 |
| NW_025323476.1 | 4099789 | 4099870 | + | 1 | LOC108061686 | E3 ubiquitin-protein ligase Nedd-4 isoform X3 |
| NW_025323476.1 | 4099789 | 4099870 | + | 1 | LOC108061686 | E3 ubiquitin-protein ligase Nedd-4 isoform X1 |
| NW_025323476.1 | 4099789 | 4099870 | + | 1 | LOC108061686 | E3 ubiquitin-protein ligase Nedd-4 isoform X4 |
| NW_025323476.1 | 4099789 | 4099870 | + | 1 | LOC108061686 | E3 ubiquitin-protein ligase Nedd-4 isoform X2 |
| NW_025323476.1 | 4099789 | 4099870 | + | 1 | LOC108061686 | E3 ubiquitin-protein ligase Nedd-4 isoform X5 |
| NW_025323476.1 | 4099789 | 4099870 | + | 1 | LOC108061686 | E3 ubiquitin-protein ligase Nedd-4 isoform X8 |
| NW_025323476.1 | 4099789 | 4099870 | + | 1 | LOC108061686 | E3 ubiquitin-protein ligase Nedd-4 isoform X9 |
| NW_025323476.1 | 4099789 | 4099870 | + | 1 | LOC108061686 | E3 ubiquitin-protein ligase Nedd-4 isoform X14 |
| NW_025323476.1 | 4099789 | 4099870 | + | 1 | LOC108061686 | E3 ubiquitin-protein ligase Nedd-4 isoform X7 |
| NW_025323476.1 | 4099789 | 4099870 | + | 1 | LOC108061686 | E3 ubiquitin-protein ligase Nedd-4 isoform X6 |
| NW_025323476.1 | 4099789 | 4099870 | + | 1 | LOC108061686 | E3 ubiquitin-protein ligase Nedd-4 isoform X10 |
| NW_025323476.1 | 4100523 | 4100561 | + | 0 | LOC108061686 | E3 ubiquitin-protein ligase Nedd-4 isoform X11 |
| NW_025323476.1 | 4100523 | 4100561 | + | 0 | LOC108061686 | E3 ubiquitin-protein ligase Nedd-4 isoform X13 |
| NW_025323476.1 | 4100523 | 4100561 | + | 0 | LOC108061686 | E3 ubiquitin-protein ligase Nedd-4 isoform X1 |
| NW_025323476.1 | 4100523 | 4100561 | + | 0 | LOC108061686 | E3 ubiquitin-protein ligase Nedd-4 isoform X3 |
| NW_025323476.1 | 4100523 | 4100561 | + | 0 | LOC108061686 | E3 ubiquitin-protein ligase Nedd-4 isoform X1 |
| NW_025323476.1 | 4100523 | 4100561 | + | 0 | LOC108061686 | E3 ubiquitin-protein ligase Nedd-4 isoform X4 |
| NW_025323476.1 | 4100523 | 4100561 | + | 0 | LOC108061686 | E3 ubiquitin-protein ligase Nedd-4 isoform X2 |
| NW_025323476.1 | 4100523 | 4100561 | + | 0 | LOC108061686 | E3 ubiquitin-protein ligase Nedd-4 isoform X5 |
| NW_025323476.1 | 4100523 | 4100561 | + | 0 | LOC108061686 | E3 ubiquitin-protein ligase Nedd-4 isoform X8 |
| NW_025323476.1 | 4100523 | 4100561 | + | 0 | LOC108061686 | E3 ubiquitin-protein ligase Nedd-4 isoform X9 |
| NW_025323476.1 | 4100523 | 4100561 | + | 0 | LOC108061686 | E3 ubiquitin-protein ligase Nedd-4 isoform X14 |
| NW_025323476.1 | 4100523 | 4100561 | + | 0 | LOC108061686 | E3 ubiquitin-protein ligase Nedd-4 isoform X7 |
| NW_025323476.1 | 4100523 | 4100561 | + | 0 | LOC108061686 | E3 ubiquitin-protein ligase Nedd-4 isoform X6 |
| NW_025323476.1 | 4100523 | 4100561 | + | 0 | LOC108061686 | E3 ubiquitin-protein ligase Nedd-4 isoform X10 |
| NW_025323476.1 | 4100633 | 4100793 | + | 0 | LOC108061686 | E3 ubiquitin-protein ligase Nedd-4 isoform X3 |
| NW_025323476.1 | 4100633 | 4100793 | + | 0 | LOC108061686 | E3 ubiquitin-protein ligase Nedd-4 isoform X10 |
| NW_025323476.1 | 4100633 | 4100802 | + | 0 | LOC108061686 | E3 ubiquitin-protein ligase Nedd-4 isoform X11 |
| NW_025323476.1 | 4100633 | 4100802 | + | 0 | LOC108061686 | E3 ubiquitin-protein ligase Nedd-4 isoform X13 |
| NW_025323476.1 | 4100633 | 4100802 | + | 0 | LOC108061686 | E3 ubiquitin-protein ligase Nedd-4 isoform X1 |
| NW_025323476.1 | 4100633 | 4100802 | + | 0 | LOC108061686 | E3 ubiquitin-protein ligase Nedd-4 isoform X1 |
| NW_025323476.1 | 4100633 | 4100802 | + | 0 | LOC108061686 | E3 ubiquitin-protein ligase Nedd-4 isoform X4 |
| NW_025323476.1 | 4100633 | 4100802 | + | 0 | LOC108061686 | E3 ubiquitin-protein ligase Nedd-4 isoform X2 |
| NW_025323476.1 | 4100633 | 4100802 | + | 0 | LOC108061686 | E3 ubiquitin-protein ligase Nedd-4 isoform X5 |
| NW_025323476.1 | 4100633 | 4100802 | + | 0 | LOC108061686 | E3 ubiquitin-protein ligase Nedd-4 isoform X8 |
| NW_025323476.1 | 4100633 | 4100802 | + | 0 | LOC108061686 | E3 ubiquitin-protein ligase Nedd-4 isoform X9 |
| NW_025323476.1 | 4100633 | 4100802 | + | 0 | LOC108061686 | E3 ubiquitin-protein ligase Nedd-4 isoform X14 |
| NW_025323476.1 | 4100633 | 4100802 | + | 0 | LOC108061686 | E3 ubiquitin-protein ligase Nedd-4 isoform X7 |
| NW_025323476.1 | 4100633 | 4100802 | + | 0 | LOC108061686 | E3 ubiquitin-protein ligase Nedd-4 isoform X6 |
| NW_025323476.1 | 4101779 | 4101929 | + | 1 | LOC108061686 | E3 ubiquitin-protein ligase Nedd-4 isoform X11 |
| NW_025323476.1 | 4101779 | 4101929 | + | 1 | LOC108061686 | E3 ubiquitin-protein ligase Nedd-4 isoform X13 |
| NW_025323476.1 | 4101779 | 4101929 | + | 1 | LOC108061686 | E3 ubiquitin-protein ligase Nedd-4 isoform X1 |
| NW_025323476.1 | 4101779 | 4101929 | + | 1 | LOC108061686 | E3 ubiquitin-protein ligase Nedd-4 isoform X1 |
| NW_025323476.1 | 4101779 | 4101929 | + | 1 | LOC108061686 | E3 ubiquitin-protein ligase Nedd-4 isoform X4 |
| NW_025323476.1 | 4101779 | 4101929 | + | 1 | LOC108061686 | E3 ubiquitin-protein ligase Nedd-4 isoform X8 |
| NW_025323476.1 | 4101779 | 4101929 | + | 1 | LOC108061686 | E3 ubiquitin-protein ligase Nedd-4 isoform X6 |
| NW_025323476.1 | 4101782 | 4101929 | + | 1 | LOC108061686 | E3 ubiquitin-protein ligase Nedd-4 isoform X3 |
| NW_025323476.1 | 4101782 | 4101929 | + | 1 | LOC108061686 | E3 ubiquitin-protein ligase Nedd-4 isoform X2 |
| NW_025323476.1 | 4101782 | 4101929 | + | 1 | LOC108061686 | E3 ubiquitin-protein ligase Nedd-4 isoform X5 |
| NW_025323476.1 | 4101782 | 4101929 | + | 1 | LOC108061686 | E3 ubiquitin-protein ligase Nedd-4 isoform X9 |
| NW_025323476.1 | 4101782 | 4101929 | + | 1 | LOC108061686 | E3 ubiquitin-protein ligase Nedd-4 isoform X14 |
| NW_025323476.1 | 4101782 | 4101929 | + | 1 | LOC108061686 | E3 ubiquitin-protein ligase Nedd-4 isoform X7 |
| NW_025323476.1 | 4101782 | 4101929 | + | 1 | LOC108061686 | E3 ubiquitin-protein ligase Nedd-4 isoform X10 |
| NW_025323476.1 | 4101991 | 4102115 | + | 0 | LOC108061686 | E3 ubiquitin-protein ligase Nedd-4 isoform X11 |
| NW_025323476.1 | 4101991 | 4102115 | + | 0 | LOC108061686 | E3 ubiquitin-protein ligase Nedd-4 isoform X13 |
| NW_025323476.1 | 4101991 | 4102115 | + | 0 | LOC108061686 | E3 ubiquitin-protein ligase Nedd-4 isoform X1 |
| NW_025323476.1 | 4101991 | 4102115 | + | 0 | LOC108061686 | E3 ubiquitin-protein ligase Nedd-4 isoform X3 |
| NW_025323476.1 | 4101991 | 4102115 | + | 0 | LOC108061686 | E3 ubiquitin-protein ligase Nedd-4 isoform X1 |
| NW_025323476.1 | 4101991 | 4102115 | + | 0 | LOC108061686 | E3 ubiquitin-protein ligase Nedd-4 isoform X4 |
| NW_025323476.1 | 4101991 | 4102115 | + | 0 | LOC108061686 | E3 ubiquitin-protein ligase Nedd-4 isoform X2 |
| NW_025323476.1 | 4101991 | 4102115 | + | 0 | LOC108061686 | E3 ubiquitin-protein ligase Nedd-4 isoform X5 |
| NW_025323476.1 | 4101991 | 4102115 | + | 0 | LOC108061686 | E3 ubiquitin-protein ligase Nedd-4 isoform X8 |
| NW_025323476.1 | 4101991 | 4102115 | + | 0 | LOC108061686 | E3 ubiquitin-protein ligase Nedd-4 isoform X9 |
| NW_025323476.1 | 4101991 | 4102115 | + | 0 | LOC108061686 | E3 ubiquitin-protein ligase Nedd-4 isoform X14 |
| NW_025323476.1 | 4101991 | 4102115 | + | 0 | LOC108061686 | E3 ubiquitin-protein ligase Nedd-4 isoform X7 |
| NW_025323476.1 | 4101991 | 4102115 | + | 0 | LOC108061686 | E3 ubiquitin-protein ligase Nedd-4 isoform X6 |
| NW_025323476.1 | 4101991 | 4102115 | + | 0 | LOC108061686 | E3 ubiquitin-protein ligase Nedd-4 isoform X10 |
| NW_025323476.1 | 4102334 | 4102433 | + | 1 | LOC108061686 | E3 ubiquitin-protein ligase Nedd-4 isoform X11 |
| NW_025323476.1 | 4102334 | 4102433 | + | 1 | LOC108061686 | E3 ubiquitin-protein ligase Nedd-4 isoform X13 |
| NW_025323476.1 | 4102334 | 4102433 | + | 1 | LOC108061686 | E3 ubiquitin-protein ligase Nedd-4 isoform X1 |
| NW_025323476.1 | 4102334 | 4102433 | + | 1 | LOC108061686 | E3 ubiquitin-protein ligase Nedd-4 isoform X3 |
| NW_025323476.1 | 4102334 | 4102433 | + | 1 | LOC108061686 | E3 ubiquitin-protein ligase Nedd-4 isoform X1 |
| NW_025323476.1 | 4102334 | 4102433 | + | 1 | LOC108061686 | E3 ubiquitin-protein ligase Nedd-4 isoform X4 |
| NW_025323476.1 | 4102334 | 4102433 | + | 1 | LOC108061686 | E3 ubiquitin-protein ligase Nedd-4 isoform X2 |
| NW_025323476.1 | 4102334 | 4102433 | + | 1 | LOC108061686 | E3 ubiquitin-protein ligase Nedd-4 isoform X5 |
| NW_025323476.1 | 4102334 | 4102433 | + | 1 | LOC108061686 | E3 ubiquitin-protein ligase Nedd-4 isoform X8 |
| NW_025323476.1 | 4102334 | 4102433 | + | 1 | LOC108061686 | E3 ubiquitin-protein ligase Nedd-4 isoform X9 |
| NW_025323476.1 | 4102334 | 4102433 | + | 1 | LOC108061686 | E3 ubiquitin-protein ligase Nedd-4 isoform X14 |
| NW_025323476.1 | 4102334 | 4102433 | + | 1 | LOC108061686 | E3 ubiquitin-protein ligase Nedd-4 isoform X7 |
| NW_025323476.1 | 4102334 | 4102433 | + | 1 | LOC108061686 | E3 ubiquitin-protein ligase Nedd-4 isoform X6 |
| NW_025323476.1 | 4102334 | 4102433 | + | 1 | LOC108061686 | E3 ubiquitin-protein ligase Nedd-4 isoform X10 |
| NW_025323476.1 | 4103205 | 4103392 | + | 0 | LOC108061686 | E3 ubiquitin-protein ligase Nedd-4 isoform X11 |
| NW_025323476.1 | 4103205 | 4103392 | + | 0 | LOC108061686 | E3 ubiquitin-protein ligase Nedd-4 isoform X13 |
| NW_025323476.1 | 4103205 | 4103392 | + | 0 | LOC108061686 | E3 ubiquitin-protein ligase Nedd-4 isoform X1 |
| NW_025323476.1 | 4103205 | 4103392 | + | 0 | LOC108061686 | E3 ubiquitin-protein ligase Nedd-4 isoform X3 |
| NW_025323476.1 | 4103205 | 4103392 | + | 0 | LOC108061686 | E3 ubiquitin-protein ligase Nedd-4 isoform X1 |
| NW_025323476.1 | 4103205 | 4103392 | + | 0 | LOC108061686 | E3 ubiquitin-protein ligase Nedd-4 isoform X4 |
| NW_025323476.1 | 4103205 | 4103392 | + | 0 | LOC108061686 | E3 ubiquitin-protein ligase Nedd-4 isoform X2 |
| NW_025323476.1 | 4103205 | 4103392 | + | 0 | LOC108061686 | E3 ubiquitin-protein ligase Nedd-4 isoform X5 |
| NW_025323476.1 | 4103205 | 4103392 | + | 0 | LOC108061686 | E3 ubiquitin-protein ligase Nedd-4 isoform X14 |
| NW_025323476.1 | 4103205 | 4103392 | + | 0 | LOC108061686 | E3 ubiquitin-protein ligase Nedd-4 isoform X7 |
| NW_025323476.1 | 4103205 | 4103392 | + | 0 | LOC108061686 | E3 ubiquitin-protein ligase Nedd-4 isoform X6 |
| NW_025323476.1 | 4103554 | 4103815 | + | 1 | LOC108061686 | E3 ubiquitin-protein ligase Nedd-4 isoform X11 |
| NW_025323476.1 | 4103554 | 4103815 | + | 1 | LOC108061686 | E3 ubiquitin-protein ligase Nedd-4 isoform X13 |
| NW_025323476.1 | 4103554 | 4103815 | + | 1 | LOC108061686 | E3 ubiquitin-protein ligase Nedd-4 isoform X1 |
| NW_025323476.1 | 4103554 | 4103815 | + | 1 | LOC108061686 | E3 ubiquitin-protein ligase Nedd-4 isoform X3 |
| NW_025323476.1 | 4103554 | 4103815 | + | 1 | LOC108061686 | E3 ubiquitin-protein ligase Nedd-4 isoform X1 |
| NW_025323476.1 | 4103554 | 4103815 | + | 1 | LOC108061686 | E3 ubiquitin-protein ligase Nedd-4 isoform X2 |
| NW_025323476.1 | 4103554 | 4103815 | + | 1 | LOC108061686 | E3 ubiquitin-protein ligase Nedd-4 isoform X6 |
| NW_025323476.1 | 4103593 | 4103815 | + | 1 | LOC108061686 | E3 ubiquitin-protein ligase Nedd-4 isoform X4 |
| NW_025323476.1 | 4103593 | 4103815 | + | 1 | LOC108061686 | E3 ubiquitin-protein ligase Nedd-4 isoform X5 |
| NW_025323476.1 | 4103593 | 4103815 | + | 1 | LOC108061686 | E3 ubiquitin-protein ligase Nedd-4 isoform X14 |
| NW_025323476.1 | 4103593 | 4103815 | + | 1 | LOC108061686 | E3 ubiquitin-protein ligase Nedd-4 isoform X7 |
| NW_025323476.1 | 4103901 | 4103984 | + | 0 | LOC108061686 | E3 ubiquitin-protein ligase Nedd-4 isoform X13 |
| NW_025323476.1 | 4103901 | 4103984 | + | 0 | LOC108061686 | E3 ubiquitin-protein ligase Nedd-4 isoform X14 |
| NW_025323476.1 | 4104484 | 4104591 | + | 0 | LOC108061686 | E3 ubiquitin-protein ligase Nedd-4 isoform X13 |
| NW_025323476.1 | 4104484 | 4104591 | + | 0 | LOC108061686 | E3 ubiquitin-protein ligase Nedd-4 isoform X1 |
| NW_025323476.1 | 4104484 | 4104591 | + | 0 | LOC108061686 | E3 ubiquitin-protein ligase Nedd-4 isoform X3 |
| NW_025323476.1 | 4104484 | 4104591 | + | 0 | LOC108061686 | E3 ubiquitin-protein ligase Nedd-4 isoform X1 |
| NW_025323476.1 | 4104484 | 4104591 | + | 0 | LOC108061686 | E3 ubiquitin-protein ligase Nedd-4 isoform X4 |
| NW_025323476.1 | 4104484 | 4104591 | + | 0 | LOC108061686 | E3 ubiquitin-protein ligase Nedd-4 isoform X2 |
| NW_025323476.1 | 4104484 | 4104591 | + | 0 | LOC108061686 | E3 ubiquitin-protein ligase Nedd-4 isoform X5 |
| NW_025323476.1 | 4104484 | 4104591 | + | 0 | LOC108061686 | E3 ubiquitin-protein ligase Nedd-4 isoform X8 |
| NW_025323476.1 | 4104484 | 4104591 | + | 0 | LOC108061686 | E3 ubiquitin-protein ligase Nedd-4 isoform X9 |
| NW_025323476.1 | 4104484 | 4104591 | + | 0 | LOC108061686 | E3 ubiquitin-protein ligase Nedd-4 isoform X14 |
| NW_025323476.1 | 4104484 | 4104591 | + | 0 | LOC108061686 | E3 ubiquitin-protein ligase Nedd-4 isoform X7 |
| NW_025323476.1 | 4104484 | 4104591 | + | 0 | LOC108061686 | E3 ubiquitin-protein ligase Nedd-4 isoform X6 |
| NW_025323476.1 | 4104484 | 4104591 | + | 0 | LOC108061686 | E3 ubiquitin-protein ligase Nedd-4 isoform X10 |
| NW_025323476.1 | 4104484 | 4104641 | + | 0 | LOC108061686 | E3 ubiquitin-protein ligase Nedd-4 isoform X11 |
| NW_025323476.1 | 4104627 | 4104641 | + | 0 | LOC108061686 | E3 ubiquitin-protein ligase Nedd-4 isoform X12 |
| NW_025323476.1 | 4104750 | 4104798 | + | 1 | LOC108061686 | E3 ubiquitin-protein ligase Nedd-4 isoform X11 |
| NW_025323476.1 | 4104750 | 4104987 | + | 0 | LOC108061686 | E3 ubiquitin-protein ligase Nedd-4 isoform X13 |
| NW_025323476.1 | 4104750 | 4104987 | + | 0 | LOC108061686 | E3 ubiquitin-protein ligase Nedd-4 isoform X1 |
| NW_025323476.1 | 4104750 | 4104987 | + | 0 | LOC108061686 | E3 ubiquitin-protein ligase Nedd-4 isoform X3 |
| NW_025323476.1 | 4104750 | 4104987 | + | 0 | LOC108061686 | E3 ubiquitin-protein ligase Nedd-4 isoform X1 |
| NW_025323476.1 | 4104750 | 4104987 | + | 0 | LOC108061686 | E3 ubiquitin-protein ligase Nedd-4 isoform X4 |
| NW_025323476.1 | 4104750 | 4104987 | + | 0 | LOC108061686 | E3 ubiquitin-protein ligase Nedd-4 isoform X2 |
| NW_025323476.1 | 4104750 | 4104987 | + | 0 | LOC108061686 | E3 ubiquitin-protein ligase Nedd-4 isoform X5 |
| NW_025323476.1 | 4104750 | 4104987 | + | 0 | LOC108061686 | E3 ubiquitin-protein ligase Nedd-4 isoform X8 |
| NW_025323476.1 | 4104750 | 4104987 | + | 0 | LOC108061686 | E3 ubiquitin-protein ligase Nedd-4 isoform X9 |
| NW_025323476.1 | 4104750 | 4104987 | + | 0 | LOC108061686 | E3 ubiquitin-protein ligase Nedd-4 isoform X14 |
| NW_025323476.1 | 4104750 | 4104987 | + | 0 | LOC108061686 | E3 ubiquitin-protein ligase Nedd-4 isoform X7 |
| NW_025323476.1 | 4104750 | 4104987 | + | 0 | LOC108061686 | E3 ubiquitin-protein ligase Nedd-4 isoform X6 |
| NW_025323476.1 | 4104750 | 4104987 | + | 0 | LOC108061686 | E3 ubiquitin-protein ligase Nedd-4 isoform X10 |
| NW_025323476.1 | 4104750 | 4104987 | + | 0 | LOC108061686 | E3 ubiquitin-protein ligase Nedd-4 isoform X12 |
| NW_025323476.1 | 4105397 | 4105521 | + | 2 | LOC108061686 | E3 ubiquitin-protein ligase Nedd-4 isoform X13 |
| NW_025323476.1 | 4105397 | 4105521 | + | 2 | LOC108061686 | E3 ubiquitin-protein ligase Nedd-4 isoform X1 |
| NW_025323476.1 | 4105397 | 4105521 | + | 2 | LOC108061686 | E3 ubiquitin-protein ligase Nedd-4 isoform X3 |
| NW_025323476.1 | 4105397 | 4105521 | + | 2 | LOC108061686 | E3 ubiquitin-protein ligase Nedd-4 isoform X1 |
| NW_025323476.1 | 4105397 | 4105521 | + | 2 | LOC108061686 | E3 ubiquitin-protein ligase Nedd-4 isoform X4 |
| NW_025323476.1 | 4105397 | 4105521 | + | 2 | LOC108061686 | E3 ubiquitin-protein ligase Nedd-4 isoform X2 |
| NW_025323476.1 | 4105397 | 4105521 | + | 2 | LOC108061686 | E3 ubiquitin-protein ligase Nedd-4 isoform X5 |
| NW_025323476.1 | 4105397 | 4105521 | + | 2 | LOC108061686 | E3 ubiquitin-protein ligase Nedd-4 isoform X8 |
| NW_025323476.1 | 4105397 | 4105521 | + | 2 | LOC108061686 | E3 ubiquitin-protein ligase Nedd-4 isoform X9 |
| NW_025323476.1 | 4105397 | 4105521 | + | 2 | LOC108061686 | E3 ubiquitin-protein ligase Nedd-4 isoform X14 |
| NW_025323476.1 | 4105397 | 4105521 | + | 2 | LOC108061686 | E3 ubiquitin-protein ligase Nedd-4 isoform X7 |
| NW_025323476.1 | 4105397 | 4105521 | + | 2 | LOC108061686 | E3 ubiquitin-protein ligase Nedd-4 isoform X6 |
| NW_025323476.1 | 4105397 | 4105521 | + | 2 | LOC108061686 | E3 ubiquitin-protein ligase Nedd-4 isoform X10 |
| NW_025323476.1 | 4105397 | 4105521 | + | 2 | LOC108061686 | E3 ubiquitin-protein ligase Nedd-4 isoform X12 |
| NW_025323476.1 | 4105590 | 4105731 | + | 0 | LOC108061686 | E3 ubiquitin-protein ligase Nedd-4 isoform X13 |
| NW_025323476.1 | 4105590 | 4105731 | + | 0 | LOC108061686 | E3 ubiquitin-protein ligase Nedd-4 isoform X1 |
| NW_025323476.1 | 4105590 | 4105731 | + | 0 | LOC108061686 | E3 ubiquitin-protein ligase Nedd-4 isoform X3 |
| NW_025323476.1 | 4105590 | 4105731 | + | 0 | LOC108061686 | E3 ubiquitin-protein ligase Nedd-4 isoform X1 |
| NW_025323476.1 | 4105590 | 4105731 | + | 0 | LOC108061686 | E3 ubiquitin-protein ligase Nedd-4 isoform X4 |
| NW_025323476.1 | 4105590 | 4105731 | + | 0 | LOC108061686 | E3 ubiquitin-protein ligase Nedd-4 isoform X2 |
| NW_025323476.1 | 4105590 | 4105731 | + | 0 | LOC108061686 | E3 ubiquitin-protein ligase Nedd-4 isoform X5 |
| NW_025323476.1 | 4105590 | 4105731 | + | 0 | LOC108061686 | E3 ubiquitin-protein ligase Nedd-4 isoform X8 |
| NW_025323476.1 | 4105590 | 4105731 | + | 0 | LOC108061686 | E3 ubiquitin-protein ligase Nedd-4 isoform X9 |
| NW_025323476.1 | 4105590 | 4105731 | + | 0 | LOC108061686 | E3 ubiquitin-protein ligase Nedd-4 isoform X14 |
| NW_025323476.1 | 4105590 | 4105731 | + | 0 | LOC108061686 | E3 ubiquitin-protein ligase Nedd-4 isoform X7 |
| NW_025323476.1 | 4105590 | 4105731 | + | 0 | LOC108061686 | E3 ubiquitin-protein ligase Nedd-4 isoform X6 |
| NW_025323476.1 | 4105590 | 4105731 | + | 0 | LOC108061686 | E3 ubiquitin-protein ligase Nedd-4 isoform X10 |
| NW_025323476.1 | 4105590 | 4105731 | + | 0 | LOC108061686 | E3 ubiquitin-protein ligase Nedd-4 isoform X12 |
| NW_025323476.1 | 4105792 | 4105948 | + | 2 | LOC108061686 | E3 ubiquitin-protein ligase Nedd-4 isoform X13 |
| NW_025323476.1 | 4105792 | 4105948 | + | 2 | LOC108061686 | E3 ubiquitin-protein ligase Nedd-4 isoform X1 |
| NW_025323476.1 | 4105792 | 4105948 | + | 2 | LOC108061686 | E3 ubiquitin-protein ligase Nedd-4 isoform X3 |
| NW_025323476.1 | 4105792 | 4105948 | + | 2 | LOC108061686 | E3 ubiquitin-protein ligase Nedd-4 isoform X1 |
| NW_025323476.1 | 4105792 | 4105948 | + | 2 | LOC108061686 | E3 ubiquitin-protein ligase Nedd-4 isoform X4 |
| NW_025323476.1 | 4105792 | 4105948 | + | 2 | LOC108061686 | E3 ubiquitin-protein ligase Nedd-4 isoform X2 |
| NW_025323476.1 | 4105792 | 4105948 | + | 2 | LOC108061686 | E3 ubiquitin-protein ligase Nedd-4 isoform X5 |
| NW_025323476.1 | 4105792 | 4105948 | + | 2 | LOC108061686 | E3 ubiquitin-protein ligase Nedd-4 isoform X8 |
| NW_025323476.1 | 4105792 | 4105948 | + | 2 | LOC108061686 | E3 ubiquitin-protein ligase Nedd-4 isoform X9 |
| NW_025323476.1 | 4105792 | 4105948 | + | 2 | LOC108061686 | E3 ubiquitin-protein ligase Nedd-4 isoform X14 |
| NW_025323476.1 | 4105792 | 4105948 | + | 2 | LOC108061686 | E3 ubiquitin-protein ligase Nedd-4 isoform X7 |
| NW_025323476.1 | 4105792 | 4105948 | + | 2 | LOC108061686 | E3 ubiquitin-protein ligase Nedd-4 isoform X6 |
| NW_025323476.1 | 4105792 | 4105948 | + | 2 | LOC108061686 | E3 ubiquitin-protein ligase Nedd-4 isoform X10 |
| NW_025323476.1 | 4105792 | 4105948 | + | 2 | LOC108061686 | E3 ubiquitin-protein ligase Nedd-4 isoform X12 |
| NW_025323476.1 | 4106010 | 4106303 | + | 1 | LOC108061686 | E3 ubiquitin-protein ligase Nedd-4 isoform X13 |
| NW_025323476.1 | 4106010 | 4106303 | + | 1 | LOC108061686 | E3 ubiquitin-protein ligase Nedd-4 isoform X1 |
| NW_025323476.1 | 4106010 | 4106303 | + | 1 | LOC108061686 | E3 ubiquitin-protein ligase Nedd-4 isoform X3 |
| NW_025323476.1 | 4106010 | 4106303 | + | 1 | LOC108061686 | E3 ubiquitin-protein ligase Nedd-4 isoform X1 |
| NW_025323476.1 | 4106010 | 4106303 | + | 1 | LOC108061686 | E3 ubiquitin-protein ligase Nedd-4 isoform X4 |
| NW_025323476.1 | 4106010 | 4106303 | + | 1 | LOC108061686 | E3 ubiquitin-protein ligase Nedd-4 isoform X2 |
| NW_025323476.1 | 4106010 | 4106303 | + | 1 | LOC108061686 | E3 ubiquitin-protein ligase Nedd-4 isoform X5 |
| NW_025323476.1 | 4106010 | 4106303 | + | 1 | LOC108061686 | E3 ubiquitin-protein ligase Nedd-4 isoform X8 |
| NW_025323476.1 | 4106010 | 4106303 | + | 1 | LOC108061686 | E3 ubiquitin-protein ligase Nedd-4 isoform X9 |
| NW_025323476.1 | 4106010 | 4106303 | + | 1 | LOC108061686 | E3 ubiquitin-protein ligase Nedd-4 isoform X14 |
| NW_025323476.1 | 4106010 | 4106303 | + | 1 | LOC108061686 | E3 ubiquitin-protein ligase Nedd-4 isoform X7 |
| NW_025323476.1 | 4106010 | 4106303 | + | 1 | LOC108061686 | E3 ubiquitin-protein ligase Nedd-4 isoform X6 |
| NW_025323476.1 | 4106010 | 4106303 | + | 1 | LOC108061686 | E3 ubiquitin-protein ligase Nedd-4 isoform X10 |
| NW_025323476.1 | 4106010 | 4106303 | + | 1 | LOC108061686 | E3 ubiquitin-protein ligase Nedd-4 isoform X12 |
| NW_025323476.1 | 4106366 | 4106764 | + | 1 | LOC108061686 | E3 ubiquitin-protein ligase Nedd-4 isoform X13 |
| NW_025323476.1 | 4106366 | 4106764 | + | 1 | LOC108061686 | E3 ubiquitin-protein ligase Nedd-4 isoform X1 |
| NW_025323476.1 | 4106366 | 4106764 | + | 1 | LOC108061686 | E3 ubiquitin-protein ligase Nedd-4 isoform X3 |
| NW_025323476.1 | 4106366 | 4106764 | + | 1 | LOC108061686 | E3 ubiquitin-protein ligase Nedd-4 isoform X1 |
| NW_025323476.1 | 4106366 | 4106764 | + | 1 | LOC108061686 | E3 ubiquitin-protein ligase Nedd-4 isoform X4 |
| NW_025323476.1 | 4106366 | 4106764 | + | 1 | LOC108061686 | E3 ubiquitin-protein ligase Nedd-4 isoform X2 |
| NW_025323476.1 | 4106366 | 4106764 | + | 1 | LOC108061686 | E3 ubiquitin-protein ligase Nedd-4 isoform X5 |
| NW_025323476.1 | 4106366 | 4106764 | + | 1 | LOC108061686 | E3 ubiquitin-protein ligase Nedd-4 isoform X8 |
| NW_025323476.1 | 4106366 | 4106764 | + | 1 | LOC108061686 | E3 ubiquitin-protein ligase Nedd-4 isoform X9 |
| NW_025323476.1 | 4106366 | 4106764 | + | 1 | LOC108061686 | E3 ubiquitin-protein ligase Nedd-4 isoform X14 |
| NW_025323476.1 | 4106366 | 4106764 | + | 1 | LOC108061686 | E3 ubiquitin-protein ligase Nedd-4 isoform X7 |
| NW_025323476.1 | 4106366 | 4106764 | + | 1 | LOC108061686 | E3 ubiquitin-protein ligase Nedd-4 isoform X6 |
| NW_025323476.1 | 4106366 | 4106764 | + | 1 | LOC108061686 | E3 ubiquitin-protein ligase Nedd-4 isoform X10 |
| NW_025323476.1 | 4106366 | 4106764 | + | 1 | LOC108061686 | E3 ubiquitin-protein ligase Nedd-4 isoform X12 |
| NW_025323476.1 | 4106822 | 4106921 | + | 1 | LOC108061686 | E3 ubiquitin-protein ligase Nedd-4 isoform X13 |
| NW_025323476.1 | 4106822 | 4106921 | + | 1 | LOC108061686 | E3 ubiquitin-protein ligase Nedd-4 isoform X1 |
| NW_025323476.1 | 4106822 | 4106921 | + | 1 | LOC108061686 | E3 ubiquitin-protein ligase Nedd-4 isoform X3 |
| NW_025323476.1 | 4106822 | 4106921 | + | 1 | LOC108061686 | E3 ubiquitin-protein ligase Nedd-4 isoform X1 |
| NW_025323476.1 | 4106822 | 4106921 | + | 1 | LOC108061686 | E3 ubiquitin-protein ligase Nedd-4 isoform X4 |
| NW_025323476.1 | 4106822 | 4106921 | + | 1 | LOC108061686 | E3 ubiquitin-protein ligase Nedd-4 isoform X2 |
| NW_025323476.1 | 4106822 | 4106921 | + | 1 | LOC108061686 | E3 ubiquitin-protein ligase Nedd-4 isoform X5 |
| NW_025323476.1 | 4106822 | 4106921 | + | 1 | LOC108061686 | E3 ubiquitin-protein ligase Nedd-4 isoform X8 |
| NW_025323476.1 | 4106822 | 4106921 | + | 1 | LOC108061686 | E3 ubiquitin-protein ligase Nedd-4 isoform X9 |
| NW_025323476.1 | 4106822 | 4106921 | + | 1 | LOC108061686 | E3 ubiquitin-protein ligase Nedd-4 isoform X14 |
| NW_025323476.1 | 4106822 | 4106921 | + | 1 | LOC108061686 | E3 ubiquitin-protein ligase Nedd-4 isoform X7 |
| NW_025323476.1 | 4106822 | 4106921 | + | 1 | LOC108061686 | E3 ubiquitin-protein ligase Nedd-4 isoform X6 |
| NW_025323476.1 | 4106822 | 4106921 | + | 1 | LOC108061686 | E3 ubiquitin-protein ligase Nedd-4 isoform X10 |
| NW_025323476.1 | 4106822 | 4106921 | + | 1 | LOC108061686 | E3 ubiquitin-protein ligase Nedd-4 isoform X12 |
| NW_025323476.1 | 4108605 | 4109163 | - | 1 | LOC108061691 | serine protease 1-like |
| NW_025323476.1 | 4109222 | 4109448 | - | 0 | LOC108061691 | serine protease 1-like |
| NW_025323476.1 | 4110646 | 4110956 | - | 2 | LOC108061644 | brachyurin |
| NW_025323476.1 | 4111269 | 4111531 | - | 1 | LOC108061644 | brachyurin |
| NW_025323476.1 | 4111590 | 4111828 | - | 0 | LOC108061644 | brachyurin |
| NW_025323476.1 | 4112686 | 4113489 | + | 0 | LOC108061690 | brachyurin |
| NW_025323476.1 | 4119524 | 4121544 | - | 2 | LOC108061714 | cyclin-T |
| NW_025323476.1 | 4121602 | 4122185 | - | 1 | LOC108061714 | cyclin-T |
| NW_025323476.1 | 4122831 | 4122876 | - | 2 | LOC108061714 | cyclin-T |
| NW_025323476.1 | 4122960 | 4123527 | - | 0 | LOC108061714 | cyclin-T |
| NW_025323476.1 | 4127980 | 4128974 | - | 2 | LOC108061715 | ecdysone-induced protein 74EF isoform X2 |
| NW_025323476.1 | 4127980 | 4128974 | - | 2 | LOC108061715 | ecdysone-induced protein 74EF isoform X1 |
| NW_025323476.1 | 4131119 | 4131373 | - | 2 | LOC108061715 | ecdysone-induced protein 74EF isoform X2 |
| NW_025323476.1 | 4131119 | 4131373 | - | 2 | LOC108061715 | ecdysone-induced protein 74EF isoform X1 |
| NW_025323476.1 | 4132037 | 4132408 | - | 2 | LOC108061715 | ecdysone-induced protein 74EF isoform X2 |
| NW_025323476.1 | 4132037 | 4132408 | - | 2 | LOC108061715 | ecdysone-induced protein 74EF isoform X1 |
| NW_025323476.1 | 4141939 | 4143004 | - | 0 | LOC108061715 | ecdysone-induced protein 74EF isoform X1 |
| NW_025323476.1 | 4166796 | 4167291 | - | 0 | LOC108061715 | ecdysone-induced protein 74EF isoform X2 |
| NW_025323476.1 | 4167496 | 4167771 | - | 0 | LOC108061715 | ecdysone-induced protein 74EF isoform X2 |
| NW_025323476.1 | 4170078 | 4170164 | - | 0 | LOC108061715 | ecdysone-induced protein 74EF isoform X2 |
| NW_025323476.1 | 4196608 | 4196667 | - | 0 | LOC108061657 | charged multivesicular body protein 5 |
| NW_025323476.1 | 4196730 | 4196960 | - | 0 | LOC108061657 | charged multivesicular body protein 5 |
| NW_025323476.1 | 4197255 | 4197420 | - | 1 | LOC108061657 | charged multivesicular body protein 5 |
| NW_025323476.1 | 4197635 | 4197786 | - | 0 | LOC108061657 | charged multivesicular body protein 5 |
| NW_025323476.1 | 4197855 | 4197923 | - | 0 | LOC108061657 | charged multivesicular body protein 5 |
| NW_025323476.1 | 4199306 | 4199496 | + | 0 | LOC108061720 | integral membrane protein GPR155 isoform X1 |
| NW_025323476.1 | 4199306 | 4199496 | + | 0 | LOC108061720 | integral membrane protein GPR155 isoform X2 |
| NW_025323476.1 | 4203325 | 4203614 | + | 1 | LOC108061720 | integral membrane protein GPR155 isoform X1 |
| NW_025323476.1 | 4203325 | 4203614 | + | 1 | LOC108061720 | integral membrane protein GPR155 isoform X2 |
| NW_025323476.1 | 4204210 | 4204349 | + | 2 | LOC108061720 | integral membrane protein GPR155 isoform X1 |
| NW_025323476.1 | 4204210 | 4204349 | + | 2 | LOC108061720 | integral membrane protein GPR155 isoform X2 |
| NW_025323476.1 | 4204412 | 4204743 | + | 0 | LOC108061720 | integral membrane protein GPR155 isoform X1 |
| NW_025323476.1 | 4204412 | 4204743 | + | 0 | LOC108061720 | integral membrane protein GPR155 isoform X2 |
| NW_025323476.1 | 4204807 | 4204972 | + | 1 | LOC108061720 | integral membrane protein GPR155 isoform X1 |
| NW_025323476.1 | 4204807 | 4204972 | + | 1 | LOC108061720 | integral membrane protein GPR155 isoform X2 |
| NW_025323476.1 | 4205038 | 4205277 | + | 0 | LOC108061720 | integral membrane protein GPR155 isoform X1 |
| NW_025323476.1 | 4205038 | 4205277 | + | 0 | LOC108061720 | integral membrane protein GPR155 isoform X2 |
| NW_025323476.1 | 4205338 | 4205529 | + | 0 | LOC108061720 | integral membrane protein GPR155 isoform X1 |
| NW_025323476.1 | 4205338 | 4205529 | + | 0 | LOC108061720 | integral membrane protein GPR155 isoform X2 |
| NW_025323476.1 | 4205595 | 4205748 | + | 0 | LOC108061720 | integral membrane protein GPR155 isoform X1 |
| NW_025323476.1 | 4205595 | 4205748 | + | 0 | LOC108061720 | integral membrane protein GPR155 isoform X2 |
| NW_025323476.1 | 4206000 | 4206068 | + | 2 | LOC108061720 | integral membrane protein GPR155 isoform X2 |
| NW_025323476.1 | 4206000 | 4206092 | + | 2 | LOC108061720 | integral membrane protein GPR155 isoform X1 |
| NW_025323476.1 | 4206149 | 4206530 | + | 2 | LOC108061720 | integral membrane protein GPR155 isoform X1 |
| NW_025323476.1 | 4206149 | 4206530 | + | 2 | LOC108061720 | integral membrane protein GPR155 isoform X2 |
| NW_025323476.1 | 4206599 | 4206973 | + | 1 | LOC108061720 | integral membrane protein GPR155 isoform X1 |
| NW_025323476.1 | 4206599 | 4206973 | + | 1 | LOC108061720 | integral membrane protein GPR155 isoform X2 |
| NW_025323476.1 | 4207029 | 4207154 | + | 1 | LOC108061720 | integral membrane protein GPR155 isoform X1 |
| NW_025323476.1 | 4207029 | 4207154 | + | 1 | LOC108061720 | integral membrane protein GPR155 isoform X2 |
| NW_025323476.1 | 4207515 | 4207713 | + | 1 | LOC108061720 | integral membrane protein GPR155 isoform X1 |
| NW_025323476.1 | 4207515 | 4207713 | + | 1 | LOC108061720 | integral membrane protein GPR155 isoform X2 |
| NW_025323476.1 | 4211949 | 4212154 | + | 0 | LOC108061704 | prostaglandin D2 receptor |
| NW_025323476.1 | 4214461 | 4214677 | + | 1 | LOC108061704 | prostaglandin D2 receptor |
| NW_025323476.1 | 4215867 | 4216059 | + | 0 | LOC108061704 | prostaglandin D2 receptor |
| NW_025323476.1 | 4216123 | 4216478 | + | 2 | LOC108061704 | prostaglandin D2 receptor |
| NW_025323476.1 | 4217857 | 4218120 | + | 0 | LOC108061704 | prostaglandin D2 receptor |
| NW_025323476.1 | 4219520 | 4221074 | - | 1 | LOC108061703 | zinc finger protein on ecdysone puffs |
| NW_025323476.1 | 4222185 | 4222414 | - | 0 | LOC108061703 | zinc finger protein on ecdysone puffs |
| NW_025323476.1 | 4224808 | 4225110 | - | 0 | LOC108061703 | zinc finger protein on ecdysone puffs |
| NW_025323476.1 | 4232211 | 4233017 | - | 0 | LOC108061705 | NEDD4 family-interacting protein 1-like |
| NW_025323476.1 | 4233803 | 4234444 | - | 0 | LOC108061706 | protein spitz |
| NW_025323476.1 | 4248516 | 4250930 | - | 0 | LOC108061708 | probable serine/threonine-protein kinase yakA |
| NW_025323476.1 | 4263374 | 4263554 | + | 0 | LOC108061665 | uncharacterized protein LOC108061665 |
| NW_025323476.1 | 4268529 | 4268701 | + | 2 | LOC108061665 | uncharacterized protein LOC108061665 |
| NW_025323476.1 | 4279758 | 4279958 | + | 0 | LOC108061665 | uncharacterized protein LOC108061665 |
| NW_025323476.1 | 4320280 | 4320372 | + | 0 | LOC108061713 | basic-leucine zipper transcription factor A |
| NW_025323476.1 | 4320474 | 4320495 | + | 0 | LOC108061713 | basic-leucine zipper transcription factor A |
| NW_025323476.1 | 4324391 | 4324579 | - | 0 | LOC108061639 | uncharacterized protein LOC108061639 |
| NW_025323476.1 | 4324639 | 4325073 | - | 0 | LOC108061639 | uncharacterized protein LOC108061639 |
| NW_025323476.1 | 4327276 | 4327355 | + | 2 | LOC108061713 | basic-leucine zipper transcription factor A |
| NW_025323476.1 | 4329064 | 4329208 | + | 0 | LOC108061713 | basic-leucine zipper transcription factor A |
| NW_025323476.1 | 4330102 | 4330183 | - | 1 | LOC108061640 | phospholipid-transporting ATPase ABCA3 |
| NW_025323476.1 | 4330330 | 4335464 | - | 0 | LOC108061640 | phospholipid-transporting ATPase ABCA3 |
| NW_025323476.1 | 4336019 | 4336308 | + | 2 | LOC108061713 | basic-leucine zipper transcription factor A |
| NW_025323476.1 | 4336709 | 4336861 | + | 0 | LOC108061713 | basic-leucine zipper transcription factor A |
| NW_025323476.1 | 4338083 | 4338352 | + | 0 | LOC108061713 | basic-leucine zipper transcription factor A |
| NW_025323476.1 | 4340071 | 4341528 | + | 0 | LOC108061711 | spermine oxidase |
| NW_025323476.1 | 4347283 | 4348722 | - | 0 | LOC108061712 | spermine oxidase |
| NW_025323476.1 | 4349543 | 4349929 | + | 0 | LOC108061713 | basic-leucine zipper transcription factor A |
| NW_025323476.1 | 4350792 | 4351388 | - | 0 | LOC108061709 | adenosine deaminase 2 |
| NW_025323476.1 | 4351755 | 4352699 | - | 0 | LOC108061709 | adenosine deaminase 2 |
| NW_025323476.1 | 4354838 | 4355461 | - | 0 | LOC108061718 | adenosine deaminase 2-A |
| NW_025323476.1 | 4355521 | 4355703 | - | 0 | LOC108061718 | adenosine deaminase 2-A |
| NW_025323476.1 | 4356247 | 4357098 | - | 0 | LOC108061718 | adenosine deaminase 2-A |
| NW_025323476.1 | 4358172 | 4358777 | - | 0 | LOC108061717 | adenosine deaminase 2 |
| NW_025323476.1 | 4358835 | 4359011 | - | 0 | LOC108061717 | adenosine deaminase 2 |
| NW_025323476.1 | 4359358 | 4360257 | - | 0 | LOC108061717 | adenosine deaminase 2 |
| NW_025323476.1 | 4362662 | 4363897 | + | 0 | LOC108061719 | uncharacterized protein LOC108061719 |
| NW_025323476.1 | 4364453 | 4364737 | + | 0 | LOC108061716 | uncharacterized protein LOC108061716 |
| NW_025323476.1 | 4364932 | 4365081 | + | 0 | LOC108061716 | uncharacterized protein LOC108061716 |
| NW_025323476.1 | 4365237 | 4365491 | + | 0 | LOC108061716 | uncharacterized protein LOC108061716 |
| NW_025323476.1 | 4365547 | 4365684 | + | 0 | LOC108061716 | uncharacterized protein LOC108061716 |
| NW_025323476.1 | 4365742 | 4365798 | + | 0 | LOC108061716 | uncharacterized protein LOC108061716 |
| NW_025323476.1 | 4365852 | 4365887 | + | 0 | LOC108061716 | uncharacterized protein LOC108061716 |
| NW_025323476.1 | 4365952 | 4366062 | + | 0 | LOC108061716 | uncharacterized protein LOC108061716 |
| NW_025323476.1 | 4366119 | 4366349 | + | 0 | LOC108061716 | uncharacterized protein LOC108061716 |
| NW_025323476.1 | 4366404 | 4366476 | + | 0 | LOC108061716 | uncharacterized protein LOC108061716 |
| NW_025323476.1 | 4366533 | 4366963 | + | 2 | LOC108061716 | uncharacterized protein LOC108061716 |
| NW_025323476.1 | 4367065 | 4367166 | + | 0 | LOC108061716 | uncharacterized protein LOC108061716 |
| NW_025323476.1 | 4385806 | 4386058 | + | 0 | LOC123002988 | uncharacterized protein LOC123002988 |
| NW_025323476.1 | 4386113 | 4386381 | + | 2 | LOC123002988 | uncharacterized protein LOC123002988 |
| NW_025323476.1 | 4386663 | 4386915 | + | 0 | LOC108061664 | uncharacterized protein LOC108061664 |
| NW_025323476.1 | 4386970 | 4387247 | + | 2 | LOC108061664 | uncharacterized protein LOC108061664 |
| NW_025323476.1 | 4407876 | 4408187 | - | 0 | LOC108061666 | period circadian protein |
| NW_025323476.1 | 4411742 | 4411770 | - | 2 | LOC108066163 | uncharacterized protein LOC108066163 isoform X2 |
| NW_025323476.1 | 4411742 | 4411770 | - | 2 | LOC108066163 | uncharacterized protein LOC108066163 isoform X1 |
| NW_025323476.1 | 4411835 | 4412101 | - | 2 | LOC108066163 | uncharacterized protein LOC108066163 isoform X2 |
| NW_025323476.1 | 4411835 | 4412101 | - | 2 | LOC108066163 | uncharacterized protein LOC108066163 isoform X1 |
| NW_025323476.1 | 4412160 | 4412369 | - | 2 | LOC108066163 | uncharacterized protein LOC108066163 isoform X2 |
| NW_025323476.1 | 4412160 | 4412463 | - | 0 | LOC108066163 | uncharacterized protein LOC108066163 isoform X1 |
| NW_025323476.1 | 4412522 | 4412525 | - | 0 | LOC108066163 | uncharacterized protein LOC108066163 isoform X2 |
| NW_025323476.1 | 4413796 | 4414079 | - | 2 | LOC108066161 | uncharacterized protein LOC108066161 |
| NW_025323476.1 | 4414133 | 4414306 | - | 2 | LOC108066161 | uncharacterized protein LOC108066161 |
| NW_025323476.1 | 4415399 | 4415489 | - | 0 | LOC108066161 | uncharacterized protein LOC108066161 |
| NW_025323476.1 | 4415562 | 4415801 | - | 0 | LOC108066161 | uncharacterized protein LOC108066161 |
| NW_025323476.1 | 4421461 | 4421569 | + | 0 | LOC108066171 | uncharacterized protein LOC108066171 |
| NW_025323476.1 | 4421624 | 4421721 | + | 2 | LOC108066171 | uncharacterized protein LOC108066171 |
| NW_025323476.1 | 4421775 | 4421958 | + | 0 | LOC108066171 | uncharacterized protein LOC108066171 |
| NW_025323476.1 | 4422608 | 4422693 | + | 2 | LOC108066171 | uncharacterized protein LOC108066171 |
| NW_025323476.1 | 4426589 | 4428001 | - | 0 | LOC108066190 | nucleobindin-2 |
| NW_025323476.1 | 4428782 | 4429018 | - | 0 | LOC108066190 | nucleobindin-2 |
| NW_025323476.1 | 4429423 | 4429753 | - | 1 | LOC108066191 | probable ATP-dependent RNA helicase DDX52 |
| NW_025323476.1 | 4429815 | 4431184 | - | 0 | LOC108066191 | probable ATP-dependent RNA helicase DDX52 |
| NW_025323476.1 | 4431239 | 4431301 | - | 0 | LOC108066191 | probable ATP-dependent RNA helicase DDX52 |
| NW_025323476.1 | 4432009 | 4432905 | - | 0 | LOC108066166 | uncharacterized protein LOC108066166 |
| NW_025323476.1 | 4433637 | 4433966 | - | 0 | LOC108066166 | uncharacterized protein LOC108066166 |
| NW_025323476.1 | 4434545 | 4434579 | - | 2 | LOC108066192 | arylsulfatase B |
| NW_025323476.1 | 4434638 | 4435534 | - | 2 | LOC108066192 | arylsulfatase B |
| NW_025323476.1 | 4436268 | 4436756 | - | 2 | LOC108066192 | arylsulfatase B |
| NW_025323476.1 | 4437248 | 4437407 | - | 0 | LOC108066192 | arylsulfatase B |
| NW_025323476.1 | 4437465 | 4437575 | - | 0 | LOC108066192 | arylsulfatase B |
| NW_025323476.1 | 4439125 | 4439556 | - | 0 | LOC108066160 | battenin |
| NW_025323476.1 | 4439619 | 4439958 | - | 1 | LOC108066160 | battenin |
| NW_025323476.1 | 4440022 | 4440180 | - | 1 | LOC108066160 | battenin |
| NW_025323476.1 | 4440295 | 4440425 | - | 0 | LOC108066160 | battenin |
| NW_025323476.1 | 4440509 | 4440718 | - | 0 | LOC108066160 | battenin |
| NW_025323476.1 | 4446361 | 4446580 | - | 1 | LOC108066156 | ionotropic receptor 75a |
| NW_025323476.1 | 4446979 | 4447259 | - | 0 | LOC108066156 | ionotropic receptor 75a |
| NW_025323476.1 | 4447315 | 4447927 | - | 1 | LOC108066156 | ionotropic receptor 75a |
| NW_025323476.1 | 4448299 | 4448446 | - | 2 | LOC108066156 | ionotropic receptor 75a |
| NW_025323476.1 | 4448498 | 4448837 | - | 0 | LOC108066156 | ionotropic receptor 75a |
| NW_025323476.1 | 4448893 | 4449077 | - | 2 | LOC108066156 | ionotropic receptor 75a |
| NW_025323476.1 | 4449137 | 4449233 | - | 0 | LOC108066156 | ionotropic receptor 75a |
| NW_025323476.1 | 4452138 | 4452653 | - | 0 | LOC108066170 | ionotropic receptor 75a |
| NW_025323476.1 | 4453227 | 4453833 | - | 1 | LOC108066170 | ionotropic receptor 75a |
| NW_025323476.1 | 4454015 | 4454151 | - | 0 | LOC108066170 | ionotropic receptor 75a |
| NW_025323476.1 | 4454208 | 4454546 | - | 0 | LOC108066170 | ionotropic receptor 75a |
| NW_025323476.1 | 4454709 | 4454887 | - | 2 | LOC108066170 | ionotropic receptor 75a |
| NW_025323476.1 | 4454944 | 4455037 | - | 0 | LOC108066170 | ionotropic receptor 75a |
| NW_025323476.1 | 4462424 | 4462865 | + | 0 | LOC108066176 | gamma-aminobutyric acid receptor alpha-like |
| NW_025323476.1 | 4464569 | 4464636 | + | 2 | LOC108066176 | gamma-aminobutyric acid receptor alpha-like |
| NW_025323476.1 | 4464984 | 4465287 | + | 0 | LOC108066176 | gamma-aminobutyric acid receptor alpha-like |
| NW_025323476.1 | 4467878 | 4467933 | - | 2 | LOC108066179 | uncharacterized protein LOC108066179 |
| NW_025323476.1 | 4470547 | 4470628 | - | 0 | LOC108066179 | uncharacterized protein LOC108066179 |
| NW_025323476.1 | 4470714 | 4471070 | - | 0 | LOC108066179 | uncharacterized protein LOC108066179 |
| NW_025323476.1 | 4475897 | 4476262 | + | 2 | LOC108066176 | gamma-aminobutyric acid receptor alpha-like |
| NW_025323476.1 | 4476444 | 4477381 | + | 2 | LOC108066176 | gamma-aminobutyric acid receptor alpha-like |
| NW_025323476.1 | 4478471 | 4478630 | - | 1 | LOC108066178 | glycerol-3-phosphate phosphatase |
| NW_025323476.1 | 4478685 | 4479372 | - | 2 | LOC108066178 | glycerol-3-phosphate phosphatase |
| NW_025323476.1 | 4480596 | 4480692 | - | 0 | LOC108066178 | glycerol-3-phosphate phosphatase |
| NW_025323476.1 | 4481041 | 4481888 | - | 2 | LOC108066177 | glycerol-3-phosphate phosphatase |
| NW_025323476.1 | 4482669 | 4482807 | - | 0 | LOC108066177 | glycerol-3-phosphate phosphatase |
| NW_025323476.1 | 4483718 | 4484187 | - | 2 | LOC108066193 | mediator of RNA polymerase II transcription subunit 19 |
| NW_025323476.1 | 4484254 | 4484368 | - | 0 | LOC108066193 | mediator of RNA polymerase II transcription subunit 19 |
| NW_025323476.1 | 4485362 | 4485799 | - | 0 | LOC108066193 | mediator of RNA polymerase II transcription subunit 19 |
| NW_025323476.1 | 4487032 | 4487073 | + | 0 | LOC108066186 | dihydrolipoyl dehydrogenase, mitochondrial |
| NW_025323476.1 | 4487367 | 4487894 | + | 0 | LOC108066186 | dihydrolipoyl dehydrogenase, mitochondrial |
| NW_025323476.1 | 4488455 | 4489333 | + | 0 | LOC108066186 | dihydrolipoyl dehydrogenase, mitochondrial |
| NW_025323476.1 | 4489491 | 4489553 | + | 0 | LOC108066186 | dihydrolipoyl dehydrogenase, mitochondrial |
| NW_025323476.1 | 4490517 | 4490738 | + | 0 | LOC108066174 | tryptophan--tRNA ligase |
| NW_025323476.1 | 4490807 | 4491231 | + | 0 | LOC108066174 | tryptophan--tRNA ligase |
| NW_025323476.1 | 4491979 | 4493329 | + | 1 | LOC108066174 | tryptophan--tRNA ligase |
| NW_025323476.1 | 4493852 | 4494300 | - | 2 | LOC108066175 | cationic amino acid transporter 2 isoform X1 |
| NW_025323476.1 | 4493852 | 4494300 | - | 2 | LOC108066175 | cationic amino acid transporter 2 isoform X2 |
| NW_025323476.1 | 4494644 | 4495429 | - | 2 | LOC108066175 | cationic amino acid transporter 2 isoform X1 |
| NW_025323476.1 | 4494644 | 4495429 | - | 2 | LOC108066175 | cationic amino acid transporter 2 isoform X2 |
| NW_025323476.1 | 4495939 | 4496199 | - | 2 | LOC108066175 | cationic amino acid transporter 2 isoform X1 |
| NW_025323476.1 | 4495939 | 4496199 | - | 2 | LOC108066175 | cationic amino acid transporter 2 isoform X2 |
| NW_025323476.1 | 4496288 | 4496654 | - | 0 | LOC108066175 | cationic amino acid transporter 2 isoform X2 |
| NW_025323476.1 | 4496288 | 4496655 | - | 1 | LOC108066175 | cationic amino acid transporter 2 isoform X1 |
| NW_025323476.1 | 4498752 | 4498777 | - | 0 | LOC108066175 | cationic amino acid transporter 2 isoform X1 |
| NW_025323476.1 | 4500586 | 4500738 | + | 0 | LOC108066157 | antigen 5 like allergen Cul n 1-like |
| NW_025323476.1 | 4500849 | 4501240 | + | 0 | LOC108066157 | antigen 5 like allergen Cul n 1-like |
| NW_025323476.1 | 4501297 | 4501727 | + | 1 | LOC108066157 | antigen 5 like allergen Cul n 1-like |
| NW_025323476.1 | 4502403 | 4502413 | + | 2 | LOC108066157 | antigen 5 like allergen Cul n 1-like |
| NW_025323476.1 | 4506413 | 4506565 | + | 0 | LOC108066159 | venom allergen 5-like |
| NW_025323476.1 | 4507208 | 4507599 | + | 0 | LOC108066159 | venom allergen 5-like |
| NW_025323476.1 | 4507656 | 4508247 | + | 1 | LOC108066159 | venom allergen 5-like |
| NW_025323476.1 | 4509010 | 4509153 | + | 0 | LOC123002989 | uncharacterized protein LOC123002989 |
| NW_025323476.1 | 4509486 | 4509868 | + | 0 | LOC123002989 | uncharacterized protein LOC123002989 |
| NW_025323476.1 | 4509924 | 4510488 | + | 1 | LOC123002989 | uncharacterized protein LOC123002989 |
| NW_025323476.1 | 4510541 | 4510750 | + | 0 | LOC123002989 | uncharacterized protein LOC123002989 |
| NW_025323476.1 | 4511506 | 4511658 | + | 0 | LOC108066167 | antigen 5 like allergen Cul n 1-like |
| NW_025323476.1 | 4512047 | 4512438 | + | 0 | LOC108066167 | antigen 5 like allergen Cul n 1-like |
| NW_025323476.1 | 4512494 | 4512995 | + | 1 | LOC108066167 | antigen 5 like allergen Cul n 1-like |
| NW_025323476.1 | 4514804 | 4514941 | + | 0 | LOC108066188 | arylsulfatase B |
| NW_025323476.1 | 4516132 | 4516291 | + | 0 | LOC108066188 | arylsulfatase B |
| NW_025323476.1 | 4516382 | 4516608 | + | 2 | LOC108066188 | arylsulfatase B |
| NW_025323476.1 | 4516830 | 4517252 | + | 0 | LOC108066188 | arylsulfatase B |
| NW_025323476.1 | 4517896 | 4518705 | + | 0 | LOC108066188 | arylsulfatase B |
| NW_025323476.1 | 4519881 | 4520350 | - | 2 | LOC108066165 | uncharacterized protein LOC108066165 |
| NW_025323476.1 | 4520412 | 4520478 | - | 0 | LOC108066165 | uncharacterized protein LOC108066165 |
| NW_025323476.1 | 4521645 | 4522199 | - | 0 | LOC108066168 | uncharacterized protein LOC108066168 |
| NW_025323476.1 | 4523249 | 4523821 | - | 0 | LOC108066164 | uncharacterized protein LOC108066164 |
| NW_025323476.1 | 4526647 | 4526757 | + | 0 | LOC108066181 | arylsulfatase B |
| NW_025323476.1 | 4526820 | 4526979 | + | 0 | LOC108066181 | arylsulfatase B |
| NW_025323476.1 | 4527046 | 4527531 | + | 2 | LOC108066181 | arylsulfatase B |
| NW_025323476.1 | 4527606 | 4527763 | + | 2 | LOC108066181 | arylsulfatase B |
| NW_025323476.1 | 4528081 | 4528899 | + | 0 | LOC108066181 | arylsulfatase B |
| NW_025323476.1 | 4529819 | 4529863 | - | 0 | LOC108066182 | CD151 antigen |
| NW_025323476.1 | 4529939 | 4530235 | - | 0 | LOC108066182 | CD151 antigen |
| NW_025323476.1 | 4530296 | 4530391 | - | 0 | LOC108066182 | CD151 antigen |
| NW_025323476.1 | 4530628 | 4530819 | - | 0 | LOC108066182 | CD151 antigen |
| NW_025323476.1 | 4530889 | 4530966 | - | 0 | LOC108066182 | CD151 antigen |
| NW_025323476.1 | 4538638 | 4539247 | - | 1 | LOC108066185 | sulfate anion transporter 1 |
| NW_025323476.1 | 4539319 | 4539467 | - | 0 | LOC108066185 | sulfate anion transporter 1 |
| NW_025323476.1 | 4539531 | 4540274 | - | 0 | LOC108066185 | sulfate anion transporter 1 |
| NW_025323476.1 | 4540475 | 4541207 | - | 1 | LOC108066185 | sulfate anion transporter 1 |
| NW_025323476.1 | 4541422 | 4541447 | - | 0 | LOC108066185 | sulfate anion transporter 1 |
| NW_025323476.1 | 4548197 | 4548471 | + | 0 | LOC108066184 | dynein axonemal assembly factor 10 |
| NW_025323476.1 | 4548529 | 4549378 | + | 1 | LOC108066184 | dynein axonemal assembly factor 10 |
| NW_025323476.1 | 4549620 | 4551075 | - | 1 | LOC108066183 | tetratricopeptide repeat protein 27 |
| NW_025323476.1 | 4551138 | 4551640 | - | 0 | LOC108066183 | tetratricopeptide repeat protein 27 |
| NW_025323476.1 | 4551697 | 4552067 | - | 2 | LOC108066183 | tetratricopeptide repeat protein 27 |
| NW_025323476.1 | 4552128 | 4552191 | - | 0 | LOC108066183 | tetratricopeptide repeat protein 27 |
| NW_025323476.1 | 4597236 | 4599644 | - | 0 | LOC108066172 | ecdysone-induced protein 75B isoform X1 |
| NW_025323476.1 | 4597236 | 4599644 | - | 0 | LOC108066172 | ecdysone-induced protein 75B, isoforms C/D isoform X4 |
| NW_025323476.1 | 4597236 | 4599644 | - | 0 | LOC108066172 | ecdysone-induced protein 75B, isoforms C/D isoform X3 |
| NW_025323476.1 | 4597236 | 4599644 | - | 0 | LOC108066172 | ecdysone-induced protein 75B isoform X2 |
| NW_025323476.1 | 4597236 | 4599644 | - | 0 | LOC108066172 | ecdysone-induced protein 75B, isoforms C/D isoform X5 |
| NW_025323476.1 | 4602962 | 4603200 | - | 2 | LOC108066172 | ecdysone-induced protein 75B isoform X1 |
| NW_025323476.1 | 4602962 | 4603200 | - | 2 | LOC108066172 | ecdysone-induced protein 75B, isoforms C/D isoform X4 |
| NW_025323476.1 | 4602962 | 4603200 | - | 2 | LOC108066172 | ecdysone-induced protein 75B, isoforms C/D isoform X3 |
| NW_025323476.1 | 4602962 | 4603200 | - | 2 | LOC108066172 | ecdysone-induced protein 75B isoform X2 |
| NW_025323476.1 | 4602962 | 4603200 | - | 2 | LOC108066172 | ecdysone-induced protein 75B, isoforms C/D isoform X5 |
| NW_025323476.1 | 4604634 | 4604697 | - | 0 | LOC108066172 | ecdysone-induced protein 75B, isoforms C/D isoform X5 |
| NW_025323476.1 | 4612836 | 4612980 | - | 0 | LOC108066172 | ecdysone-induced protein 75B isoform X1 |
| NW_025323476.1 | 4612836 | 4612980 | - | 0 | LOC108066172 | ecdysone-induced protein 75B, isoforms C/D isoform X4 |
| NW_025323476.1 | 4612836 | 4612980 | - | 0 | LOC108066172 | ecdysone-induced protein 75B, isoforms C/D isoform X3 |
| NW_025323476.1 | 4612836 | 4612980 | - | 0 | LOC108066172 | ecdysone-induced protein 75B isoform X2 |
| NW_025323476.1 | 4613390 | 4614565 | - | 0 | LOC108066172 | ecdysone-induced protein 75B isoform X2 |
| NW_025323476.1 | 4638665 | 4638753 | - | 2 | LOC108066172 | ecdysone-induced protein 75B isoform X1 |
| NW_025323476.1 | 4638665 | 4638753 | - | 2 | LOC108066172 | ecdysone-induced protein 75B, isoforms C/D isoform X4 |
| NW_025323476.1 | 4638665 | 4638753 | - | 2 | LOC108066172 | ecdysone-induced protein 75B, isoforms C/D isoform X3 |
| NW_025323476.1 | 4644474 | 4645173 | - | 0 | LOC108066172 | ecdysone-induced protein 75B, isoforms C/D isoform X3 |
| NW_025323476.1 | 4673261 | 4673345 | - | 0 | LOC108066172 | ecdysone-induced protein 75B, isoforms C/D isoform X4 |
| NW_025323476.1 | 4708168 | 4709524 | - | 0 | LOC108066172 | ecdysone-induced protein 75B isoform X1 |
| NW_025323476.1 | 4238853 | 4238983 | + | 0 | LOC108061707 | selenoprotein F |
| NW_025323476.1 | 4503831 | 4503983 | + | 0 | LOC108066158 | antigen 5 like allergen Cul n 1-like |
| NW_025323476.1 | 4239578 | 4239953 | + | 1 | LOC108061707 | selenoprotein F |
| NW_025323476.1 | 4504087 | 4504478 | + | 0 | LOC108066158 | antigen 5 like allergen Cul n 1-like |
| NW_025323476.1 | 4504535 | 4505051 | + | 1 | LOC108066158 | antigen 5 like allergen Cul n 1-like |
| NW_025323476.1 | 4401400 | 4401485 | + | 0 | LOC108061647 | LOW QUALITY PROTEIN: elongation of very long chain fatty acids protein 4-like |
| NW_025323476.1 | 4405053 | 4405148 | + | 1 | LOC108061647 | LOW QUALITY PROTEIN: elongation of very long chain fatty acids protein 4-like |
| NW_025323476.1 | 4405344 | 4405506 | + | 1 | LOC108061647 | LOW QUALITY PROTEIN: elongation of very long chain fatty acids protein 4-like |
| NW_025323476.1 | 4406127 | 4406253 | + | 0 | LOC108061647 | LOW QUALITY PROTEIN: elongation of very long chain fatty acids protein 4-like |
| NW_025323476.1 | 4406256 | 4406297 | + | 2 | LOC108061647 | LOW QUALITY PROTEIN: elongation of very long chain fatty acids protein 4-like |
| NW_025323476.1 | 4406359 | 4406657 | + | 2 | LOC108061647 | LOW QUALITY PROTEIN: elongation of very long chain fatty acids protein 4-like |
| NW_025323476.1 | 3671791 | 3672230 | + | 0 | LOC108055239 | LOW QUALITY PROTEIN: uncharacterized protein LOC108055239 |
| NW_025323476.1 | 3673960 | 3674297 | + | 1 | LOC108055239 | LOW QUALITY PROTEIN: uncharacterized protein LOC108055239 |
| NW_025323476.1 | 3674788 | 3675460 | + | 2 | LOC108055239 | LOW QUALITY PROTEIN: uncharacterized protein LOC108055239 |
| NW_025323476.1 | 3675697 | 3675718 | + | 1 | LOC108055239 | LOW QUALITY PROTEIN: uncharacterized protein LOC108055239 |
| NW_025323476.1 | 3696681 | 3697349 | - | 0 | LOC108055267 | LOW QUALITY PROTEIN: zinc finger protein 776 |
| NW_025323476.1 | 3698230 | 3698790 | - | 0 | LOC108055267 | LOW QUALITY PROTEIN: zinc finger protein 776 |
| NW_025323476.1 | 4449398 | 4449605 | - | 1 | LOC108066169 | LOW QUALITY PROTEIN: ionotropic receptor 75a |
| NW_025323476.1 | 4450006 | 4450887 | - | 1 | LOC108066169 | LOW QUALITY PROTEIN: ionotropic receptor 75a |
| NW_025323476.1 | 4450939 | 4451075 | - | 0 | LOC108066169 | LOW QUALITY PROTEIN: ionotropic receptor 75a |
| NW_025323476.1 | 4451132 | 4451473 | - | 0 | LOC108066169 | LOW QUALITY PROTEIN: ionotropic receptor 75a |
| NW_025323476.1 | 4451529 | 4451695 | - | 2 | LOC108066169 | LOW QUALITY PROTEIN: ionotropic receptor 75a |
| NW_025323476.1 | 4451753 | 4451843 | - | 0 | LOC108066169 | LOW QUALITY PROTEIN: ionotropic receptor 75a |
