# Supplementary material for: A male-killing Wolbachia endosymbiont is concealed by another endosymbiont and a nuclear suppressor
Source: PLoS Biol. 2023 Mar 22;21(3):e3001879. doi: 10.1371/journal.pbio.3001879 (PMC10069767; doi:10.1371/journal.pbio.3001879)
Supplement: Attachment — Submitted filename: reviews and responses-final.docx [file pbio.3001879.s011.docx]

REVIEWERS' COMMENTS *(with responses in italics)*

Reviewer #1:

This paper is a holistic organismal investigation of the parameters that influence the existence of a double symbiont infection in flies in which the symbionts are CI-Wolbachia and MK-Wolbachia. I found the investigation largely comprehensive and the insights relevant to those studying MK in natural and laboratory populations. This paper stands on its own for documenting the co-existence of CI and MK Wolbachia combined with a suite of studies that contextualize this observation. While many individual pieces of data are not new, the combined approaches are a solid standard for the field to think about. Please find below my major and minor comments, all of which are important to update in the revision.

Major comments.

1. This paper by Hurst et al. seems to be essential for the introduction and discussion of the paper, the latter of which could be more deeply expanded as it felt too short for such a wonderful set of results.

Which Way to Manipulate Host Reproduction? Wolbachia That Cause Cytoplasmic Incompatibility Are Easily Invaded by Sex Ratio-Distorting Mutants by Hurst et al.

*We are certainly aware of this paper and now mention it and its main conclusion relevant to the current study both in the Introduction (line 46) and Discussion (line 426).*

2. The abstract should mention the presence of wmk in the MK Wolbachia for contexual, genetic relevance to the field and potential genetic underpinnings of the trait. This makes the work more interesting to the rapidly developing area.

*Added at line 31.*

3. Add branch lengths to the phylogeny. Move the phylogeny earlier into the results for evolutionary reference and context relative to the rest of the study.

*We have updated the figure to include branch lengths. The new figure and legend are now presented as Figure S1, and are referenced earlier in the manuscript as requested by the reviewer.*

**Figure S1.** An estimated Bayesian phylogram for various Group-A (red) and Group-B (blue) *Wolbachia* strains. *w*Pse *Smith+* is a Group-A strain and outgroup to a larger clade containing *w*Ha, *w*Ri-like, and *w*Mel-like strains. The four *Wolbachia* infecting *Nomada* bees (*w*NFe, *w*NPa, *w*NLeu, and *w*NFa) are outgroup to the clade containing *w*Pse *Smith+*. These Group-A *Wolbachia* diverged from Group-B *Wolbachia* (*w*Pip_Pel and *w*No) up to 46MYA (divergence time superimposed from Meany et al. [28]). The phylogram was estimated with 168 genes and a total of 136,545 bp. All nodes have posterior probability support of 1. Branch lengths are reported, with their sum fixed to one. Very small branch lengths (i.e., =/< 0.003) are excluded to improve figure readability.

4. Make a wmk and cifA phylogeny. Add other wmk homologs that do not kill males here too into the wmk tree. See below for related paper and wMel alleles.

*We now present phylogenies of cifA_[T1]_, cifA_[T2]_, and wmk. The cifA phylogenies leverage data from Martinez et al. (2021, MBE), and the wmk phylogeny leverages data from Permutter et al. (2019, PP) as suggested by the reviewer. We have also taken the opportunity to characterize WO prophage present in our assemblies using the serine recombinase locus, as described in Bordenstein and Bordenstein (2022, PLoS Genetics).*

*cifA*_[T1]_ phylogeny:

**Figure S3. An estimated Bayesian phylogram for various *cifA*_[T1]_ copies.** Identical samples were collapsed into a single tip, and nodes with posterior probability <0.95 were collapsed into polytomies. The root position is not known, but the tree was midpoint rooted for legibility. *cifA_wPse_N101[2]_* is identical to *cifA_wPse_Smith+_* and sister to the *cifA*_[T1]_ set observed in the genome of the unnamed *Wolbachia* variant that infects gall wasp *Biorhiza pallida*. *cifA_wPse_N101[1]_*, *cifA_wMel_*, *cifA_wRec_*, *cifA_wSan_*, *cifA_wTeis_*, and *cifA_wYak_* comprise a polytomy that is sister to a clade containing *cifA_wHa_* and *cifA_wSh_*, assuming that the true root does not fall within this focal clade. We only report the posterior probability node support values that are less than 1.

*cifA*_[T2]_ phylogeny:

**Figure S4. An estimated Bayesian phylogram for various *cifA*_[T2]_ copies.** Identical samples were collapsed into a single tip, and nodes with posterior probability <0.95 were collapsed into polytomies. The root position is not known, but the tree was midpoint rooted for legibility. *cifA_wPse_Smith+_* and *cifA_wPse_N101_* copies are identical and sister to *cifA_wBic._* from *Drosophila bicornuta*. assuming the true root does not fall in this clade. All nodes shown have a posterior probability support value of 1.

*wmk* phylogeny:

**Figure S5. An estimated Bayesian phylogram for *wmk* copies characterised in this study added to the analysis presented in Perlmutter et al. (2019).** Identical samples were collapsed into a single tip, and nodes with posterior probability <0.95 were collapsed into polytomies. The root position is not known, but the tree was midpoint rooted for legibility. Node support values < 1 are denoted.

5. The quote from the paper (See below) is only partially true since mortality is elevated throughout development, but there is liekly embryonic lethality when comparing the CI control cross of 95% egg to larvae hatching versus several of the other crosses that show 80% egg to larvae hatching (MK female x CI male and CI x CI). Non-parametric ANOVA statistics for small sample sizes can be completed on all of these crosses and mortality stages to reveal where the effects.

"This suggests that male killing is occurring, but at a later time point than expected based on studies in other species (e.g. D. pandora, [6])."

*We’ve added non-parametric tests across the 5 treatments for hatch rate which was marginally non- significant and other traits which were significant and we also changed the wording a bit to reflect the reviewer comment (line 151 paragraph and on).*

6. Rapid loss of MK section is very interesting, but it lacks a clear explanation in its current form. Im very curious about it. I see three crucial questions to be addressed in this section and the discussion. Add for assisting readers to think more broadly about this discovery.

(a) What are the reasons for which this may occur so rapidly in the lab? What is failing in the MK strain to cause MK? Does lab acclimation or rearing media/conditions hide MK for some reason? I don't know.

*This result led us to test for nuclear based suppression, perhaps this was not clear in this section, so we now refer more directly to it at lines 201-204. And the rapid change in the nuclear suppression phenotype in the lab was demonstrated in Fig. 3. We appreciate that there might be other factors that also contribute to this effect such as fitness benefits/costs from MK and suppression due to the lab environment, but the main driving factor in the lines that we then investigated in detail seems to have been the evolution of suppression which we then explore further.*

(b) Doesn't this phenomena also explain why MK could be relatively less common than other phenotype such as CI? Strains from the field that are maintained in the lab may hide the actual frequency of MK, if MK is not readily measured right away.

*This is an interesting idea, though we are not sure there is evidence for it given that we have found MK before in other species that are stable in the lab (cited). Again, we note that suppression seems to be involved in the present case at least in the strains examined in detail, see above. However we do now mention this briefly in the discussion (line 472 – 474).*

(c) Related to (b), Doesn't this phenomena also explain why some Wolbachia with MK genetic ability, like wMel, also not show MK in the lab? It struck me as an important, potential explanation for why wMel contains the wmk gene, but does not elicit MK.

*See above, it is possible and might be worth exploring further given the presence of wmk in wMel although we are still not convinced of the impact of this gene within a natural setting. However please note that in the original work (now largely forgotten) on melanogaster Wolbachia published in 1988 (in Entomologia experimentalis et applicata) by one of us on (what turned out to be) wMel we characterized lines based on offspring production in controlled crosses directly from the field and never saw evidence for MK (otherwise pairwise male-female based lines would not have established). You can also check the detailed sex ratio information on p 63 of that paper which is now cited. Subsequent work has confirmed this. As above, we mention this in passing but in the absence of direct support we want to be cautious.*

7. wmk in N101: it is highly interesting, though not emphasized enough in this version of the paper, that the genetic evidence for the MK strain N101 yields near complete wmk identity to the male-killing wmk gene in wMel, whereas the CI strain wmk sequences are quite divergent. The authors can highlight this association in the results and enhanced discussion section on wmk. Also, they can evaluate if the CI wmk copies are more related to wmk-related sequences tested that did not yield MK in Perlmutter et al. 2019, mSystems, "See section on Divergent wmk homologs in wMel do not…" with divergent wmk homologous genes 508, 622, 623, and 255 that did not yield MK. Notably that study found only wmk from wMel is hared across all MK genomes to date, and it is the only one that kills males by transgenic expression in Dmel.

*We agree with the reviewer that we should spend more time on this topic in the manuscript (see response to point 8). We now present a wmk phylogeny, placing our copies phylogenetically among those in Perlmutter et al. (2019). Our wmk phylogram places the unique wmk copy that we attribute to the MK-causing strain sister to the homologs in wMel, wAna, wHa, wRi, wSuz and wRec. The wmk copies in the CI-causing strain that appear pseudogenized are sister to the homologs in wBor and wInn. These three are widely diverged from the others on this tree, as noted by the reviewer.*

*Our tree is very similar to that in Perlmutter et al. (2019), with the exception of the specific placement of wBor and wInn. We attribute this to the long-root problem; that is, when there are several closely related samples and a substantially more distant sample, it is difficult to determine where among the similar samples the branch to the distant sample should attach. The distant sample is about equally distant from all of the others, making placement difficult. We collapsed wAu_0691 together with wAu_0252 and wWil since they are identical at all non-gap bases in the alignment; Perlmutter et al. (2019) also found wAu_0691 together with wBor and wInn, which is hard for us to resolve given the very long branch separating wBor and wInn from the rest of the tree. We also find lower posterior probabilities for many nodes than Perlmutter et al. (2019), causing us to collapse these into polytomies. Despite these few differences, our results provide insight into the placement of wmk alleles present in our assemblies, and are mostly in agreement with the broader relationships presented in Perlmutter et al. (2019). We now include this as Figure S5.*

8. The last sentence on page 12 strikes a tone that should be more balanced. e.g, "reflects anything about the genetic basis of MK in nature. Not only is wmk from wMel the only version of wmk shared across all MK genomes (some natural), but it is the distinguishing and causal gene of MK between related moth species that vary in MK or no MK. <https://www.biorxiv.org/content/10.1101/2022.06.12.495854v2>. It also seems probable or at least noteworth based on the sequence evidence presented here that the close wMel relative of wmk in the MK strain is the causal factor.

*We agree with the reviewer that we could have done a better job balancing this statement. We have decided to eliminate the specific text mentioned above from the manuscript. As noted above, we now present a wmk phylogeny that enables us to clearly show and discuss the very close relationship between the wmk copy we attribute to the MK-causing N101 strain and wmk from wMel, as well as the putatively pseudogenized copies in the CI-causing strain.*

*We thank the reviewer for bringing the preprint above to our attention. To balance our discussion, we now discuss both support for and against wmk’s central role in MK. For example, we highlight transgenic MK results and note the similarity in DNA defects observed in transgenic and natural systems. We also highlight the complexity here, by noting that wmk overexpression in transgenic systems often kills both males and females. To add breadth to our discussion, we also now highlight recent results on the Oscar locus that exclusively kills males in Ostrinia furnacalis (lines 453-6). We think that these additions in combination with our discussion of other possible pathogenic effects has improved the manuscript.*

Minor comments:

1. Mention the other reasons for how/why MK symbionts spread (e.g., horizontal transfer of Wolbachia by dead siblings, resource reallocation, etc) here "such as through the avoidance of sib mating" in the intro.

*Agreed, added (line 43)*

2. Mention wmk presence and divergence here as a key result and for contextual relevance as readers will wonder about wmk in particular since it is the MK candidate supported by multiple genetic and host system studies (Flies and Moths): "We use molecular approaches to characterize the MK strain which differs for some genomic regions to the coinhabiting CI strain but is identical in other regions"

*Done (line 90)*

3. More information in T2 legend will help clarify if the loss/reduction of MK in F10 generation (From F4) is due to backcrossing with a non-MK strain or waning of the MK phenotype in laboratory-reared line. That's an important difference.

*Done and clarified in the legend.*

4. Under "Genomic analysis...." section, Smith+ is first mentioned here. What is this? Please add context.

*We have provided information, it was mentioned in the methods which of course are at the bottom of the paper, but we have now pointed to Table S3 as well in the Results where this line is first mentioned.*

5. Are the cif types between Smith+ and N101 idential or not. Please mention in the results.

*Yes. We now present cifA phylogenies and new text that presents this result in detail.*

6. At "Our analyses also discovered..." Qualify what wmk is when it is first mentioned in results so reader knows the gene.

*Done*

Nice study and kudos!

*Thanks!*

Reviewer #2:

All maternally-transmitted microbes that are successful in the long range in a population have to manipulate the reproductive biology of their hosts by favoring infected females for their own spreading and maintenance, or attenuate by taking over mutualistic functions ranging from higher host fitness, fecundity, pathogen protection or behavior. As a general but pivotal problem, however, rare endosymbiotic variants with the capacity to induce significant host phenotypes like sex ratios can be easily overlooked because they are overshadowed by dominating or even fixed variants in multi-infected hosts. Hence, we most likely only see currently only the tip of the iceberg and miss the full repertoire of microbes, strains and variants affecting that can affect host phenotypes.

In this manuscript, the authors have uncovered a male-killing (MK) Wolbachia strain variant in Drosophila pseudotakahashii flies that only works in combination with the fixed CI-inducing variant and not on its own. Interestingly, doubly-infected males mated to uninfected females induce strong CI, but when mated to mono-infected females they induce MK. This is a novel and exciting observation that will expand our conceptual understanding on multi-symbiont variant interactions and their population dynamics within an individual host. In addition, different to most MK Spiroplasma or Wolbachia doubly-infected males die later at their larval and not embryonic stage, which is quite unusual and will need further investigations on its mechanism - see below.

By comparative genomics they found 17 diagnostic genes that are duplicated in the MK variant compared to the CI strain. It is a petty, however, the authors could not generate the complete MK genome of N101 with high confidence, which seems to be a technical issue since it is not feasible to separate both infections at the moment. Thus, the authors assume a double infection but unfortunately cannot provide full evidence. Did the authors try to isolate DNA from late and dying male larvae assuming the potential dominance of the MK variant over the CI variant at this stage? Hence, it would be worth checking ratios by diagnostic qPCR at different developmental stages in both sexes. Possibly, host-directed titer and/or tropism control of the MK variant via autophagy is less efficient in male larvae. Finally, can we rule out that Wolbachia is diploid? Alternatively, similar to the pathogenic wMel-Pop variant in D. melanogaster, this variant could over-replicate selectively in male larval tissues but not females explaining their late sex-specific mortality. The authors should at least consider and discuss these possibilities in the light of wMelPop and wMelCS and other potential explanations.

*We’ve done our best to outline the results indicating the presence of a double infection based on the genomic data and the data outlined in the section starting at line 141. We’d have to discard a lot of detailed genomic information in the literature to argue for Wolbachia not being a circular haploid genome! We have now made an attempt to identify larvae at the stage where death occurs by dissecting larvae from media across time but unfortunately the visual approach to sexing larvae is not going to work because the male larvae die at an early instar stage before they can be sexed. This means that we would need to develop a molecular Y chromosome marker for this species which will take substantial time and resources. This is certainly something worth doing for the future and we added it in the Discussion (lines 461-464). And we have now mentioned the other strains and their pathogenicity as suggested by the reviewer.*

Finally, in response to the cost of rare males in natural populations the emergence of nuclear MK-suppressor was already earlier described in different insect associations, but their genetic basis and molecular mechanisms are still quite unclear or rudimentary. In this study, they isolated a by elegant backcrossing a dominant nuclear suppressor region of app. 1 Mb size that emerged in the lab spontaneously by having the capacity to rescued the MK phenotype, although the variant was still present. Future studies will hopefully uncover the actual responsive gene(s) in more detail.

*Thanks!*

Although the MK strain is transmitted maternally at high fidelity but not complete in the lab, it is found only at very low frequency in nature at 4%, that is a quite common prevalence for also other MK endosymbionts of insects. In future experiments - and not this current study since not the main focus here - it would be interesting to test if single infected females avoid doubly infected males in nature or in controlled mate choice assays, which would point to behavioral suppression/avoidance or multi-mating.

*We agree – but we have to start somewhere!*

To sum up this study uncovers a new and exciting symbiotic model system to deepen our understanding on the complexity and genetic dynamics of both or even more partners on phenotype expression.

Reviewer #3:

This is an interesting account of i) presence of male-killing in a Drosophila as a coinfection ii) presence of suppression of male-killing that spreads through mixed culture from rare. The key messages that we derive are: a) PCR based screens for male-killers would fail in the presence of coinfection with similar symbionts that don't kill males. b) Male-killing can act at the larval stage in Drosophila c) Suppression is polymorphic in this system d) Suppression sweeps rapidly in laboratory mixed culture.

I think these are all well substantiated conclusions. I think c) and d) are probably the most exciting outcomes - in particular polymorphic suppression within a population is rather rare and presents opportunities for onward study. I'd argue a) was particular to Wolbachia male-killers with Wolbachia coinfection (heterospecific combinations would come out fine) - and to PCR screening for male-killers via sex biased presence, rather than phenotype based screening; thus whilst the point is well made and well taken, it is perhaps not the key driver to publish this in PloS Biology. b) (late male-killing) is interesting, but known for other male-killers (viral male-killing in tea tortrix moths, Spiroplasma in hemiiptera, and also Wolbachia introgression in Drosophila).

Important matters that may require some additional experimental work:

1. Whilst I found the case for coinfection of two similar Wolbachia, one CI and one MK, to be well made, I felt the approach to genomic analysis of Wolbachia difference to be suboptimal. Yes, we see read depth changes that imply deleted regions, but the approach can't find gain of function compared to the single infection. Use of longer read technologies (either nanopore or PacBio) would enable this complete analysis. I felt the complete analysis was important as it would allow a) precise definition of the genome for the MK strain b) given the depth of background for this strain type, a good capacity to examine potential male-killing factors. It may also be possible to do this without extra sequencing: to extract novel regions belonging to the MK with bioinformatic approaches e.g blobtools identifying Wolbachia elements and then subtracting the known genome?

*We agree with the reviewer that finely resolving the genetic basis of MK is an important question. We also agree that it may be possible to eventually resolve the complexities of the co-infected line with more data. We have leveraged long-read sequencing for the Smith+ and N101 assemblies. Since there are many regions longer than even our long reads can sequence in a single read that are shared by the two Wolbachia, we have been unable to resolve or circularize the two genomes. This is similar to how Illumina-only assemblies are fragmented by smaller repeated regions. Blobtools can identify which contigs are Wolbachia and which are host associated, but it fails to differentiate the two very similar Wolbachia. Notably, we have now identified cifs, putative WO prophages, and homologs to the wMel wmk gene in the MK strain via subtracting the Smith+ copies from the N101 assembly. Additional analyses are not currently possible without additional functional data that would provide the information needed to extend this approach to other regions of the genome potentially underlying in these traits. Also sequencing of singly infected genotypes will be required to provide a precise definition of the genome for the MK strain, but we are uncertain as to whether such a strain could be isolated and we think that is well beyond the scope of the current paper.*

Important matters that require some extra analyses and modifications in text.

2. Having defined a genomic region for suppression, it seems straightforward to improve the reach of this paper to include a map of this region and predicted genes (even if onward analysis is left for a later paper).

*We’ve now provided supplementary information that gives a list of the genes in this region (Table S4. This seems more useful than providing a map for future reference given that quite a lot of genes are involved.*

3. The results of the PCR screen for the MK Wolbachia find a consistent association of presence with male-killing from field samples, which implies suppression is rare (or not expressed in flies taken from the natural environment). I felt this rarity deserved more contrast and discussion - in an ideal world this would be a population genomic screen of the population to ascertain the frequency of the variant associated with suppression, but that would be perhaps beyond this manuscript. Nevertheless, Further discussion of why rare would be worthwhile (usual theory explanation is suppression is costly in absence of MK so you get a polymorphic equilibrium).

*This overlaps partly with the comment from another reviewer, see above. We agree that it would be really interesting to investigate the costs of the suppressor and we’ve added more discussion around this issue and rarity at lines 466-495. The system described here may well turn out to be an excellent system to investigate such issues.*

Things I think would improve the manuscript but can be considered discretionary.

i) I found the abstract rather a hard read - needs to think about the non-specialist a bit. I could read it precisely after reading the manuscript but found a bit hard as a standalone. The first sentence is true but not perhaps the main point of the manuscript.

*We’ve done some rewording to the abstract.*

ii) Intro paragraph 2 - late male-killing also known in insects e.g. <https://doi.org/10.1093/jhered/est052>

*Thanks we have added this reference.*

iii) Page 4. Suppression allows CI to be revealed (as males now survive and express the CI) rather than evolve (although it does evolve phenotypically, it did not genetically).

*Changes made to reflect this.,*

iv) Table 1 could be tested for heterogeneity between samples (I suspect no evidence to reject homogeneity as frequency is low - but worth doing). Also, the text around this table doesn't quite mirror the data in the table in terms of total N and number of lines?

*We have now added a contingency analysis, and checked the number of lines which are correct, we now refer to Table S3 for detailed information and have indicated that one region includes several populations from which low numbers were collected.*

v) I'd probably move figure 2 to the supplementary as not essential to the more high profile points being made.

*Moved*

vi) Page 18 'genetic construct' sits awkwardly as has a meaning in functional genetics as an ectopic modification. Rephrase?

*Reworded*

vii) P23 worth noting that suppression has been observed to spread readily in the field - so the lab here mirrors nature. See studies of both butterflies and lacewings.

*We’ve added references to this research.*

viii) Polymorphic male-killer suppression is known in one other case, which should be referenced at some point. 10.1371/journal.ppat.1000987

*Great, we’ve now referenced this, we were not aware of it.*

Finally, this manuscript wasn't a very easy read in some place, partly because experiments are complex (fine) but also a bit because I think there are a lot of lab-specific names that the outside reader spends some mental energy remembering and working through. It was worth the effort to do so, but would suggest that the authors look at this to improve the clarity for the reader. e.g on page 11 we are introduced to Smith + but really have no introduction to this strain prior to this.

*Apologies for this, we have now gone through it carefully to ensure that lab specific names are minimised or defined more clearly, such as Smith+ (see above). We did define the names in Table S3 and have now referred to this table more specifically in places.*

__________________________________________________
