# Supplementary material for: A male-killing Wolbachia endosymbiont is concealed by another endosymbiont and a nuclear suppressor
Source: PLoS Biol. 2023 Mar 22;21(3):e3001879. doi: 10.1371/journal.pbio.3001879 (PMC10069767; doi:10.1371/journal.pbio.3001879)
Supplement: Attachment — Submitted filename: Dear Editor.docx [file pbio.3001879.s012.docx]

Dear Editor,

Please find the manuscript with final DOIs in figure legends as requested, the updated title suggested, and final corrections to the references.

Thanks very much

Ary Hoffmann (on behalf of the authors).
